# Association of Systemic Lupus Erythematosus With Decreased Immunosuppressive Potential of the IgG Glycome

**DOI:** 10.1002/art.39273

**Published:** 2015-10-28

**Authors:** Frano Vučković, Jasminka Krištić, Ivan Gudelj, Maria Teruel, Toma Keser, Marija Pezer, Maja Pučić‐Baković, Jerko Štambuk, Irena Trbojević‐Akmačić, Clara Barrios, Tamara Pavić, Cristina Menni, Youxin Wang, Yong Zhou, Liufu Cui, Haicheng Song, Qiang Zeng, Xiuhua Guo, Bernardo A. Pons‐Estel, Paul McKeigue, Alan Leslie Patrick, Olga Gornik, Tim D. Spector, Miroslav Harjaček, Marta Alarcon‐Riquelme, Mariam Molokhia, Wei Wang, Gordan Lauc

**Affiliations:** ^1^ Genos Ltd., Glycoscience Research LaboratoryZagrebCroatia; ^2^ Pfizer–University of Granada–Junta de Andalucia Centre for Genomics and Oncological Research (GENYO)GranadaSpain; ^3^ University of ZagrebZagrebCroatia; ^4^ King's College London, London, UK, and Hospital del Mar and Institut Mar d'Investigacions MediquesBarcelonaSpain; ^5^ King's College LondonLondonUK; ^6^ Capital Medical UniversityBeijingChina; ^7^ Beijing Tiantan Hospital and Capital Medical UniversityBeijingChina; ^8^ Affiliated Kailuan General Hospital of Hebei United UniversityTangshanChina; ^9^ International Medical Center and Chinese People's Liberation Army General HospitalBeijingChina; ^10^ Sanatorio ParqueRosarioArgentina; ^11^ University of EdinburghEdinburghUK; ^12^ Kavanagh Street Medical CentrePort of SpainTrinidadWest Indies; ^13^ Clinical Hospital Center Sestre MilosrdniceZagrebCroatia; ^14^ Pfizer–University of Granada–Junta de Andalucia Centre for Genomics and Oncological Research (GENYO), Granada, Spain, and Oklahoma Medical Research FoundationOklahoma City; ^15^ Capital Medical University, Beijing, China, and Edith Cowan UniversityPerthWestern AustraliaAustralia; ^16^ Genos Ltd., Glycoscience Research Laboratory, and University of ZagrebZagrebCroatia

## Abstract

**Objective:**

Glycans attached to the Fc portion of IgG are important modulators of IgG effector functions. Interindividual differences in IgG glycome composition are large and they associate strongly with different inflammatory and autoimmune diseases. *IKZF1*, *HLA–DQ2A/B*, and *BACH2* genetic loci that affect IgG glycome composition show pleiotropy with systemic lupus erythematosus (SLE), indicating a potentially causative role of aberrant IgG glycosylation in SLE. We undertook this large multicenter case–control study to determine whether SLE is associated with altered IgG glycosylation.

**Methods:**

Using ultra‐performance liquid chromatography analysis of released glycans, we analyzed the composition of the IgG glycome in 261 SLE patients and 247 matched controls of Latin American Mestizo origin (the discovery cohort) and in 2 independent replication cohorts of different ethnicity (108 SLE patients and 193 controls from Trinidad, and 106 SLE patients and 105 controls from China).

**Results:**

Multiple statistically significant differences in IgG glycome composition were observed between patients and controls. The most significant changes included decreased galactosylation and sialylation of IgG (which regulate proinflammatory and antiinflammatory actions of IgG) as well as decreased core fucose and increased bisecting *N*‐acetylglucosamine (which affect antibody‐dependent cell‐mediated cytotoxicity).

**Conclusion:**

The IgG glycome in SLE patients is significantly altered in a way that decreases immunosuppressive action of circulating immunoglobulins. The magnitude of observed changes is associated with the intensity of the disease, indicating that aberrant IgG glycome composition or changes in IgG glycosylation may be an important molecular mechanism in SLE.

*N*‐glycans attached to the Fc portion of IgG are important modulators of IgG effector functions 1, 2 (see Supplementary Figure 1, available on the *Arthritis & Rheumatology* web site at http://onlinelibrary.wiley.com/doi/10.1002/art.39273/abstract). Glycans that lack terminal galactose activate complement and make IgG proinflammatory, while the addition of galactose decreases the inflammatory potential of IgG 1, 3. Further extension of IgG glycans by the addition of sialic acid dramatically changes the physiologic role of IgG, converting it from a proinflammatory agent into an antiinflammatory agent. This relatively small fraction of sialylated IgG is believed to be responsible for the immunosuppressive activity of intravenous immunoglobulins (IVIGs) 4. Another feature of the IgG glycan, fucose attached to the glycan core (core fucose), interferes with binding of IgG to Fcγ receptor IIIa (FcγRIIIa) and greatly diminishes its capacity for the activation of antibody‐dependent cell‐mediated cytotoxicity (ADCC) 5. The removal of core fucose from IgG glycans increases clinical efficacy of monoclonal antibodies, enhancing their therapeutic effect through ADCC‐mediated killing 6, 7. Our recent genome‐wide association study (GWAS) of the IgG glycome in 2,247 individuals identified 16 genetic loci that are associated with variations in composition of the IgG glycome 8. Three of these 16 genes (*IKZF1*, *BACH2*, and *HLA–DQA2*) have previously also been identified as GWAS hits in systemic lupus erythematosus (SLE) 9, 10, 11.

SLE is a chronic multiorgan autoimmune disease that predominantly affects women and certain ethnic groups including African/African Caribbean, American Indian, Asian Indian, Polynesian, and Chinese populations. Development of SLE includes multiple genetic and environmental risk factors that result in loss of tolerance and development of an autoreactive immune response, including autoimmune cells producing pathogenic autoantibodies, mainly of the IgG1 and IgG3 subclasses. The molecular mechanisms leading to SLE are unknown, but mice lacking α‐mannosidase II (αM‐II) develop an SLE‐like syndrome 12. Absence of αM‐II leads to the absence of complex branched *N*‐glycans 13. Studies by Green et al ruled out the notion that an ontogenic defect of the kidneys is involved in SLE pathogenesis and, by bone marrow reconstitution experiments, also excluded a role of bone marrow–derived cells. However, mice that are deficient in recombination‐activating genes and that therefore cannot generate functional B and T cells showed a more severe disease, indicating the importance of the immunosuppressive role of IgG in SLE. This idea was confirmed by the finding that IgG administration dampened SLE‐like symptoms 12. Aiming to investigate the potential role of IgG glycosylation in SLE, we analyzed IgG glycome composition in 3 cohorts of SLE patients and matching controls.

## PATIENTS AND METHODS

### Description of patient cohorts

A group of 261 SLE patients and 247 age‐, sex‐, and ethnicity‐matched controls from the Latin American Genoma de Lupus Eritematoso Sistemico Network (GENLES) study was selected for the present study. This group of patients has been extensively described 14. All patients fulfilled the American College of Rheumatology (ACR) 1982 revised criteria for SLE 15.

The data set from Trinidad used in this analysis comprised 108 SLE patients (10 male and 98 female) and 193 age‐, sex‐, and ethnicity‐matched controls with complete age, sex, and glycan data. The case definition of SLE was based on the ACR 1982 revised criteria applied to medical records (available for >90% of the patients) and to the medical history given by the patient. For the purpose of identifying patients in this study, a person was considered to have SLE if any 4 or more of the 11 elements of the criteria were present, serially or simultaneously, during any interval of observation. The study was approved by the London School of Hygiene and Tropical Medicine ethics board and the Trinidad and Tobago Ministry of Health (see ref. 16 for further details).

A total of 106 SLE patients (7 male and 99 female, ages 14–74 years) and 105 age‐ and sex‐matched controls of Han Chinese ethnicity from Northern China (Beijing and Tangshan) were included in the study. The Systemic Lupus International Collaborating Clinics revision of the ACR criteria for SLE 17 was used to diagnose SLE in this cohort. Individuals diagnosed as having cancer or specific severe diseases involving the cardiovascular system, respiratory system, genitourinary system, or digestive system were excluded. Permissions for conduct of the study were obtained from the local ethics committees, and written informed consent was obtained from all participants.

### Analysis of IgG glycans

IgG was isolated, and Fc and Fab glycans were released and analyzed by hydrophilic interaction chromatography–ultra‐performance liquid chromatography (UPLC) as described recently 18.

### Statistical analysis

Clinical characteristics among patients and controls in all 3 cohorts were compared using Wilcoxon and Fisher's exact tests. In order to remove experimental variation from measurements, normalization and batch correction were performed on UPLC glycan data. To make measurements across samples comparable, normalization by total area was performed in which the peak area of each of 24 glycan structures (glycan peaks [GPs]) was divided by the total area of the corresponding chromatogram. Prior to batch correction, normalized glycan measurements were log‐transformed due to right skewness of their distributions and the multiplicative nature of batch effects. Batch correction was performed on log‐transformed measurements using the ComBat method (R package “sva”; https://bioconductor.org/packages/sva/), in which the technical source of variation (which sample was analyzed on which plate) was modeled as a batch covariate. To get measurements corrected for experimental noise, estimated batch effects were subtracted from log‐transformed measurements.

In addition to 24 directly measured glycan structures, 57 derived traits were calculated from the directly measured glycans. These derived traits average particular glycosylation features (galactosylation, fucosylation, sialylation) across different individual glycan structures, and consequently they are more closely related to individual enzymatic activities and underlying genetic polymorphisms. As derived traits represent ratios and sums of initial glycans, they were calculated using normalized and batch‐corrected glycan measurements after transformation to the proportions (exponential transformation of batch‐corrected measurements).

Analyses of associations between disease status and glycan traits were performed using a logistic regression model with age and sex included as additional covariates. Intracase analysis was also performed in which associations between glycan levels and clinical traits (antinuclear antibody [ANA] positivity, pericarditis, proteinuria, disease duration, and the like) were examined using a regression model adjusted for age and sex, with the glycan trait described as the dependent variable. Both case–control and intracase analyses were first performed for each cohort separately and then combined using an inverse variance–weighted meta‐analysis approach (R package “metafor”; http://www.metafor-project.org/). Prior to analyses, glycan variables were all transformed to a standard normal distribution (mean = 0, SD = 1) by inverse transformation of ranks to normality (R package “GenABEL,” function rntransform; http://www.genabel.org/). Using rank‐transformed variables in case–control and intracase analyses makes estimated effects of different glycans in different cohorts comparable, as transformed glycan variables have the same standardized variance. In case–control logistic regression analysis, estimated odds ratios (ORs) always correspond to 1 SD change in the measured glycan trait. In intracase regression analysis, coefficients of binary predictors (ANA positivity, pericarditis, proteinuria) refer to change in a glycan variable between 2 classes of binary predictors expressed in SDs. The false discovery rate (FDR) for both analyses was controlled using the Benjamini‐Hochberg procedure 19, and *P* values corrected for multiple testing (with FDR set at 0.05) are shown throughout.

For prediction of SLE status, a regularized logistic (elastic net) regression model was applied (R package “glmnet”; http://www.jstatsoft.org/v33/i01/). For classification, only 24 initial glycan traits were used as predictors. Prior to model training, elastic net regularization parameters (alpha and lambda) were tuned on 50% of the Latin American cohort (250 samples), and optimal parameters chosen by the tuning procedure (α = 0, λ = 0.1) were used in all further classification models (R package “caret”; http://github.com/topepo/caret/). To evaluate the biomarker potential of glycans, the model was trained and validated on each lupus cohort separately. For each cohort 2 models were built—1 using only age and sex as predictors and 1 using age, sex, and 24 glycan traits. To evaluate performance of the predictive model based on glycans, a 10–cross‐validation procedure was used. The predictions from each validation round were merged into one validation set on which the performance of a cohort‐specific model was evaluated based on area under the curve (AUC) criteria. Additionally, the predictive power of each individual glycan trait was evaluated by receiver operating characteristic (ROC) curve analysis. Differences in glycomes between patients and controls were visualized using principal components analysis (PCA). PCA was applied only on 4 glycan variables (GP6, GP9, GP10, GP14) that showed strong predictive power in all 3 cohorts. The AUCs of different classification models were compared using a bootstrap test. Data were analyzed and visualized using R programming language (version 3.0.1).

## RESULTS

IgG glycome composition was analyzed in 261 SLE patients of Latin American Mestizo origin and 247 matched controls recruited through a multicenter collaboration 20. The first replication cohort consisted of 108 SLE patients and 193 matched controls of African Caribbean ethnicity from Trinidad. The second replication cohort consisted of 106 SLE patients and 105 matched controls of Han Chinese ethnicity from Beijing and Tangshan. Basic descriptions of the cohorts are provided in Supplementary Table 1, available on the *Arthritis & Rheumatology* web site at http://onlinelibrary.wiley.com/doi/10.1002/art.39273/abstract.

IgG glycosylation analysis was performed using a recently developed high‐throughput analysis method 21 that reliably separates and individually quantifies nearly all IgG glycans. In addition to direct measurement of 24 chromatographic peaks, an additional 57 derived traits (representing composite traits such as galactosylation, sialylation, and the like) were calculated as described previously 21.

### Galactosylation of IgG

**Figure 1:**
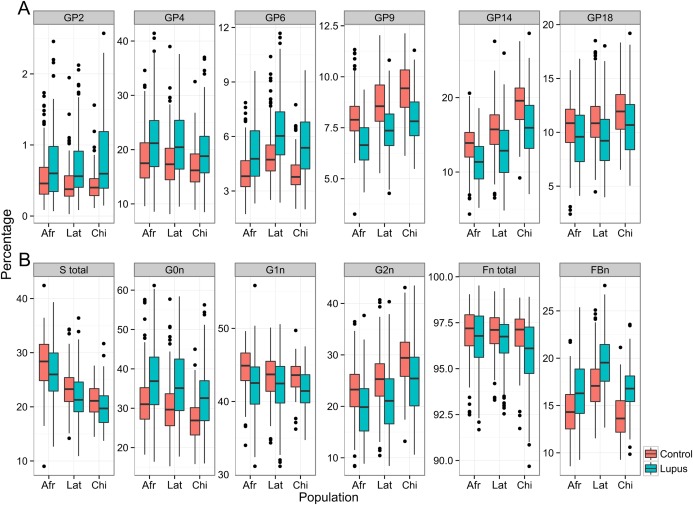
Differences in IgG glycosylation in patients with systemic lupus erythematosus and in healthy controls in 3 different populations. The IgG glycome was analyzed using hydrophilic interaction chromatography–ultra‐performance liquid chromatography in African Caribbeans (Afr; 108 patients and 193 controls), Latin Americans of Mestizo ethnicity (Lat; 261 patients and 247 controls), and Han Chinese (Chi; 106 patients and 105 controls). Pronounced differences were observed between patients and controls in directly measured glycan structures (**A**) and in derived traits that measure sialylation, galactosylation, fucosylation, and bisecting *N*‐acetylglucosamine (GlcNAc) (**B**). Data are shown as box plots. Each box represents the 25th to 75th percentiles. Lines inside the boxes represent the median. Lines outside the boxes represent the 10th and 90th percentiles. Circles indicate outliers. Glycan peak 2 (GP2) = percentage of A2 glycan in total IgG glycans; GP4 = percentage of FA2 glycan in total IgG glycans; GP6 = percentage of FA2B glycan in total IgG glycans; GP9 = percentage of FA2[3]G1 glycan in total IgG glycans; GP14 = percentage of FA2G2 glycan in total IgG glycans; GP18 = percentage of FA2G2S1 glycan in total IgG glycans; S total = proportion of sialylated structures in total IgG glycans; G0n = proportion of agalactosylated structures in neutral glycans; G1n = proportion of monogalactosylated structures in neutral glycans; G2n = proportion of digalactosylated structures in neutral glycans; Fn total = proportion of fucosylated structures in neutral glycans; FBn = proportion of fucosylated (with bisecting GlcNAc) structures in total neutral IgG glycans. Color figure can be viewed in the online issue, which is available at http://onlinelibrary.wiley.com/doi/10.1002/art.39273/abstract.

Glycans without galactose (GP1–GP6) make IgG proinflammatory by promoting complement activation 1, 3. The proportion of IgG molecules that carry agalactosylated glycans increases with age 22 and in a number of different inflammatory diseases 23. In the discovery cohort we observed a statistically significant and considerable increase in all agalactosylated glycans and in derived trait G0 (which combines all agalactosylated structures). In parallel, glycans with 2 galactoses (such as GP14) decreased significantly. The same pattern of changes was also observed in both replication SLE cohorts (Figure 1 and Tables 1 and 2) (also see Supplementary Table 2, available on the *Arthritis & Rheumatology* web site at http://onlinelibrary.wiley.com/doi/10.1002/art.39273/abstract).

**Table 1:** Associations of the directly measured glycans with disease status (SLE), adjusted for age and sex^a^

Glycan	Description	Meta‐analysis	
		OR (95% CI)	*P*
GP1	Percentage of FA1 glycan in total IgG glycans	1.93 (1.66–2.24)	1.88 × 10^−17^
GP2	Percentage of A2 glycan in total IgG glycans	2.05 (1.75–2.40)	5.40 × 10^−18^
GP4	Percentage of FA2 glycan in total IgG glycans	1.81 (1.55–2.11)	4.33 × 10^−14^
GP5	Percentage of M5 glycan in total IgG glycans	1.34 (1.17–1.53)	3.00 × 10^−5^
GP6	Percentage of FA2B glycan in total IgG glycans	3.00 (2.49–3.62)	7.38 × 10^−30^
GP7	Percentage of A2G1 glycan in total IgG glycans	1.56 (1.35–1.79)	1.47 × 10^−9^
GP8	Percentage of FA2[6]G1 glycan in total IgG glycans	0.86 (0.75–0.97)	2.40 × 10^−2^
GP9	Percentage of FA2[3]G1 glycan in total IgG glycans	0.27 (0.22–0.33)	2.08 × 10^−39^
GP10	Percentage of FA2[6]BG1 glycan in total IgG glycans	2.00 (1.72–2.32)	1.02 × 10^−18^
GP11	Percentage of FA2[3]BG1 glycan in total IgG glycans	1.10 (0.96–1.26)	1.80 × 10^−1^
GP12	Percentage of A2G2 glycan in total IgG glycans	1.19 (1.05–1.36)	1.11 × 10^−2^
GP14	Percentage of FA2G2 glycan in total IgG glycans	0.37 (0.31–0.44)	9.74 × 10^−27^
GP15	Percentage of FA2BG2 glycan in total IgG glycans	0.97 (0.84–1.11)	6.50 × 10^−1^
GP16	Percentage of FA2G1S1 glycan in total IgG glycans	0.61 (0.53–0.71)	1.63 × 10^−11^
GP17	Percentage of A2G2S1 glycan in total IgG glycans	1.22 (1.06–1.39)	5.28 × 10^−3^
GP18	Percentage of FA2G2S1 glycan in total IgG glycans	0.55 (0.47–0.64)	1.23 × 10^−13^
GP19	Percentage of FA2BG2S1 glycan in total IgG glycans	1.31 (1.14–1.49)	1.01 × 10^−4^
GP21	Percentage of A2G2S2 glycan in total IgG glycans	0.91 (0.80–1.04)	1.87 × 10^−1^
GP22	Percentage of A2BG2S2 glycan in total IgG glycans	1.59 (1.38–1.83)	1.11 × 10^−10^
GP23	Percentage of FA2G2S2 glycan in total IgG glycans	0.61 (0.53–0.70)	1.63 × 10^−11^
GP24	Percentage of FA2BG2S2 glycan in total IgG glycans	1.60 (1.40–1.84)	3.29 × 10^−11^

a SLE = systemic lupus erythematosus; OR = odds ratio; 95% CI = 95% confidence interval; GP1 = glycan peak 1.

**Table 2:** Associations of the derived glycan traits with disease status (SLE), adjusted for age and sex^a^

Description	Glycan	Meta‐analysis	
		OR (95% CI)	*P*
Proportion of agalactosylated structures in neutral glycans	G0n	2.31 (1.95–2.72)	3.14 × 10^−22^
Proportion of monogalactosylated structures in neutral glycans	G1n	0.56 (0.48–0.64)	8.42 × 10^−15^
Proportion of digalactosylated structures in neutral glycans	G2n	0.46 (0.39–0.55)	2.67 × 10^−19^
Proportion of fucosylated structures in neutral glycans	Fn total	0.67 (0.59–0.77)	1.62 × 10^−8^
Proportion of fucosylation in agalactosylated structures	FG0 total/G0	0.65 (0.56–0.74)	1.73 × 10^−9^
Proportion of fucosylated structures with bisecting GlcNAc in monogalactosylated structures	FG1 total/G1	0.61 (0.53–0.70)	1.63 × 10^−11^
Proportion of fucosylated structures with bisecting GlcNAc in digalactosylated structures	FG2 total/G2	0.51 (0.44–0.59)	7.99 × 10^−18^
Proportion of sialylated structures in total IgG glycans	S total	0.69 (0.60–0.79)	3.13 × 10^−7^
Proportion of monosialylated structures in total IgG glycans	S1 total	0.59 (0.51–0.69)	1.63 × 10^−11^
Proportion of disialylated structures in total IgG glycans	S2 total	1.00 (0.88–1.14)	9.99 × 10^−1^
Proportion of fucosylated (with bisecting GlcNAc) structures in total neutral IgG glycans	FBn	2.67 (2.25–3.17)	2.06 × 10^−28^
Proportion of fucosylation (without bisecting GlcNAc) of agalactosylated structures	FBG0/G0	1.57 (1.36–1.80)	4.71 × 10^−10^
Proportion of fucosylation (without bisecting GlcNAc) of monogalactosylated structures	FBG1/G1	2.37 (2.02–2.78)	3.00 × 10^−25^
Proportion of fucosylation (without bisecting GlcNAc) of digalactosylated structures	FBG2/G2	2.97 (2.48–3.55)	1.06 × 10^−31^
Incidence of bisecting GlcNAc in all fucosylated monosialylated structures in total IgG glycans	FBS1/(FS1 + FBS1)	2.03 (1.73–2.38)	2.23 × 10^−17^
Incidence of bisecting GlcNAc in all fucosylated disialylated structures in total IgG glycans	FBS2/(FS2 + FBS2)	4.03 (3.29–4.93)	2.39 × 10^−40^

a SLE = systemic lupus erythematosus; OR = odds ratio; 95% CI = 95% confidence interval; GlcNAc = *N*‐acetylglucosamine.

Interestingly, a significant difference was observed in 2 monogalactosylated glycan structures. Galactose can be attached to the 3‐arm and/or 6‐arm of the IgG glycan. Structures with galactose attached to the 6‐arm (GP8) are more abundant on human IgG, and only this structure decreases with age, while structures with galactose on the 3‐arm do not 22. In this study we observed a significant decrease of IgG glycan with galactose on the 3‐arm (GP9) in both the discovery and replication cohorts (Figure 1 and Table 1). Differences in biologic functions of these 2 structures are not known, and it is also not known how this type of difference can be generated since the same enzyme adds galactose to both arms. However, a recent IgG glycome GWAS identified a specific association between *BACH2* and 1 of the 2 possible monogalactosylated forms of the IgG glycan 8.

### Sialylation of IgG

The addition of sialic acid to IgG glycans converts IgG into an antiinflammatory molecule 24. Initially it was thought that only a small fraction of IgG molecules bear sialylated glycans, but our recent large study revealed that this was a methodologic artifact and that ∼50% of all digalactosylated IgG glycans are also sialylated 18. A new method that reliably quantifies IgG sialylation was used in the present study, and it enabled us to demonstrate that major sialylated glycans (GP16, GP18, and GP23) were significantly decreased in both the discovery and replication cohorts (Figure 1 and Table 1), indicating significantly decreased immunosuppressive potential of IgG in SLE. Interestingly, in contrast to major sialylated IgG glycans, sialylated glycans GP17, GP19, and GP24 were increased in SLE patients. A common characteristic of these glycans is the presence of bisecting *N*‐acetylglucosamine (GlcNAc), and the increase of this structural feature in SLE (see below) apparently outweighed the effects of a decrease in sialylation.

### Core fucose and bisecting GlcNAc

The addition of fucose to the innermost GlcNAc (core fucose) significantly attenuates binding of IgG to FcγRIIIa and serves as a safety switch that prevents ADCC 25. The addition of bisecting GlcNAc was reported to have an opposite effect and cause higher affinity for FcγRIIIa 26, but it is not clear whether this is a direct effect or an indirect consequence of decreased core fucose level, because the presence of bisecting GlcNAc inhibits the addition of core fucose 27.

We observed a significantly decreased core fucose level and a consistent increase of all UPLC GPs that contain bisecting GlcNAc in both the discovery and replication cohorts (Figure 1 and Tables 1 and 2). A derived trait that measures total core fucose (Fn total) was also significantly decreased, but the decrease in fucosylation was much more evident when this trait was decomposed into Fn (all glycans with core fucose that do not contain bisecting GlcNAc), which was decreased in SLE, and FBn (all glycans with core fucose that contain bisecting GlcNAc), which was increased in SLE.

In general, all major glycans that contain bisecting GlcNAc (GP6, GP10, GP19, and GP24) increased significantly, but the increase was more pronounced in derived traits that measure bisecting GlcNAc (Table 2). The largest observed difference between patients and controls (OR 4.03, *P* = 2.39 × 10^−40^) was in the incidence of bisecting GlcNAc in all fucosylated disialylated structures in total IgG glycans (FBS2/[FS2 + FBS2]) (Table 2).

### Association of changes in glycans with the presence of ANAs and the symptom profile

**Figure 2:**
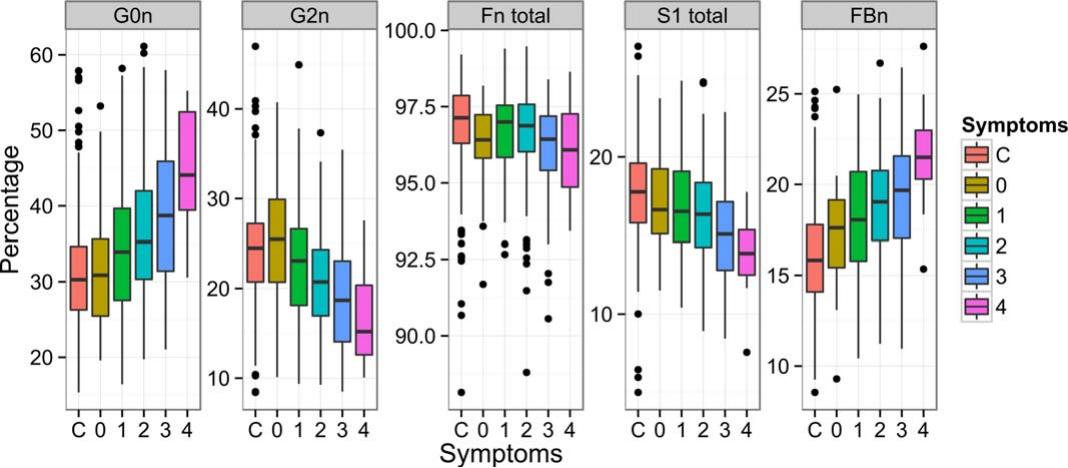
Changes in glycans are associated with symptom profile of systemic lupus erythematosus (SLE) in African Caribbean and Latin American cohorts. Prior to analysis, the 2 cohorts were pooled (n = 358). Patients were divided into groups by the number of SLE complications/symptoms. For this analysis, only symptoms that showed the strongest associations with changes in glycome composition were selected (antinuclear antibody positivity, pericarditis, proteinuria, and disease duration >8 years). Glycosylation changes were more pronounced in patients with a larger number of complications/symptoms (for G0n, *P* = 1.28 × 10^−7^; for G2n, *P* = 1.21 × 10^−8^; for Fn total, *P* = 5.27 × 10^−2^; for proportion of monosialylated structures in total IgG glycans [S1 total], *P* = 2.14 × 10^−6^; for FBn, *P* = 1.49 × 10^−6^). Data are shown as box plots. Each box represents the 25th to 75th percentiles. Lines inside the boxes represent the median. Lines outside the boxes represent the 10th and 90th percentiles. Circles indicate outliers. C = control samples (see Figure 1 for other definitions). Color figure can be viewed in the online issue, which is available at http://onlinelibrary.wiley.com/doi/10.1002/art.39273/abstract.

In addition to being different between SLE patients and controls, levels of IgG glycans were significantly different between patients with less severe disease and those with more severe disease. The presence of ANAs at the time of sampling for this study and a number of secondary phenotypes, e.g., pericarditis and proteinuria, were consistently associated with decreased galactosylation, decreased sialylation, and increased levels of bisecting GlcNAc (Table 3) (also see Supplementary Figure 2 and Supplementary Table 3, available on the *Arthritis & Rheumatology* web site at http://onlinelibrary.wiley.com/doi/10.1002/art.39273/abstract). The decrease in levels of core fucose in patients with the same phenotypes was also statistically significant, but the magnitude of significance was lower. The difference in glycome composition seemed to be cumulative, since a greater number of symptoms/complications (that were associated with changes in glycome composition) within an individual patient was associated with more extensive changes in IgG glycome composition (Figure 2).

**Table 3:** Associations between individual IgG glycans and SLE diagnostic parameters^a^

Glycan	Description	Trait	Effect, mean ± SEM	Meta‐analysis, adjusted *P*
FBS2/(FS2 + FBS2)	Bisecting GlcNAc	ANA positivity	0.67 ± 0.11	2.53 × 10^−6^
GP9	Galactosylation	ANA positivity	−0.59 ± 0.12	1.65 × 10^−4^
FBG2/(FG2 + FBG2)	Bisecting GlcNAc	ANA positivity	0.51 ± 0.11	1.00 × 10^−3^
FBn	Bisecting GlcNAc	ANA positivity	0.50 ± 0.11	1.00 × 10^−3^
FG1/G1	Fucosylation	ANA positivity	−0.51 ± 0.11	1.02 × 10^−3^
GP23	Sialylation	ANA positivity	−0.50 ± 0.12	1.86 × 10^−3^
GP6	Agalactosylation	ANA positivity	0.43 ± 0.11	4.40 × 10^−3^
FG2/G2	Fucosylation	ANA positivity	−0.38 ± 0.11	2.38 × 10^−2^
GP20	Sialylation	ANA positivity	−0.40 ± 0.12	2.38 × 10^−2^
GP14	Galactosylation	ANA positivity	−0.35 ± 0.11	3.01 × 10^−2^
G0n	Agalactosylation	ANA positivity	0.34 ± 0.11	4.13 × 10^−2^
GP10	Bisecting GlcNAc	Duration >8 years	0.29 ± 0.05	1.73 × 10^−4^
FBn	Bisecting GlcNAc	Duration >8 years	0.27 ± 0.05	4.94 × 10^−4^
FBG1/G1	Bisecting GlcNAc	Duration >8 years	0.26 ± 0.05	5.96 × 10^−4^
FBS2/(FS2 + FBS2)	Bisecting GlcNAc	Duration >8 years	0.22 ± 0.05	3.74 × 10^−3^
FG0/G0	Fucosylation	Duration >8 years	−0.19 ± 0.05	2.23 × 10^−2^
GP6n	Agalactosylation	Duration >8 years	0.16 ± 0.05	4.48 × 10^−2^
GP20	Sialylation	Duration >8 years	−0.17 ± 0.06	5.59 × 10^−2^
GP14	Galactosylation	Pericarditis	−0.50 ± 0.12	3.11 × 10^−3^
GP6	Agalactosylation	Pericarditis	0.46 ± 0.12	6.67 × 10^−3^
G2n	Galactosylation	Pericarditis	−0.44 ± 0.12	1.02 × 10^−2^
G0n	Agalactosylation	Pericarditis	0.44 ± 0.13	1.46 × 10^−2^
FG2/G2	Fucosylation	Pericarditis	−0.43 ± 0.13	2.22 × 10^−2^
GP18	Sialylation	Pericarditis	−0.38 ± 0.12	3.78 × 10^−2^
GP9n	Galactosylation	Pericarditis	−0.39 ± 0.13	4.48 × 10^−2^
GP4	Agalactosylation	Pericarditis	0.36 ± 0.13	5.60 × 10^−2^
GP2	Agalactosylation	Proteinuria	0.43 ± 0.10	1.57 × 10^−3^
FG0 total/G0	Fucosylation	Proteinuria	−0.36 ± 0.10	1.81 × 10^−2^
GP6	Agalactosylation	Proteinuria	0.31 ± 0.10	3.69 × 10^−2^
GP9n	Galactosylation	Proteinuria	−0.32 ± 0.10	4.05 × 10^−2^
FG1 total/G1	Fucosylation	Proteinuria	−0.31 ± 0.10	4.48 × 10^−2^
FG2/G2	Fucosylation	Proteinuria	−0.30 ± 0.10	4.84 × 10^−2^
GP18	Sialylation	Proteinuria	−0.28 ± 0.10	5.28 × 10^−2^

a ANA = antinuclear antibody; GP9 = glycan peak 9; FBG2/(FG2+FBG2)=incidence of bisecting GlcNAc in all fucosylated digalactosylated structures in total neutral IgG glycans; FG1/G1 = percentage of fucosylation of monogalactosylated structures; FG2/G2 = percentage of fucosylation of digalactosylated structures; FG0/G0 = percentage of fucosylation of agalactosylated structures (see Description column of Table 2 for other definitions).

### Effects of corticosteroids on IgG glycans

One potential problem for the interpretation of the observed differences was the fact that the majority of patients were receiving corticosteroids, while controls were not. Therefore, it was difficult to exclude the possibility that the observed changes in IgG glycome composition were the effect of treatment with corticosteroids. Effects of steroids on the IgG glycome are not known, but 1 recently reported study showed effects of medication (including steroids) on the total plasma glycome 28. Neutral plasma glycans originate predominantly from IgG 29; thus, by analyzing the reported data, we were able to estimate effects of corticosteroids on the IgG glycome. The only IgG glycan that was measured as a separate high‐performance liquid chromatography peak was G0, and effects of corticosteroids on it have not been reported 28.

To explore this further, we analyzed effects of steroids on a population of twins from the TwinsUK cohort who underwent IgG glycome analysis in a recent study 22. Data on steroid use were available for 2,179 twins of European ethnicity and indicated that 53 of them were receiving steroid therapy. Random intercept logistic regressions were applied to study the association of steroid use with IgG glycome composition. All models were adjusted for age, body mass index, sex, and family relatedness. After correction for multiple testing, no significant differences were observed. Since we could not identify any effects of steroids on major glycans that differed between SLE patients and controls, we concluded that the observed differences likely resulted from SLE and not from corticosteroid therapy.

### Classification of SLE using IgG glycans

**Figure 3:**
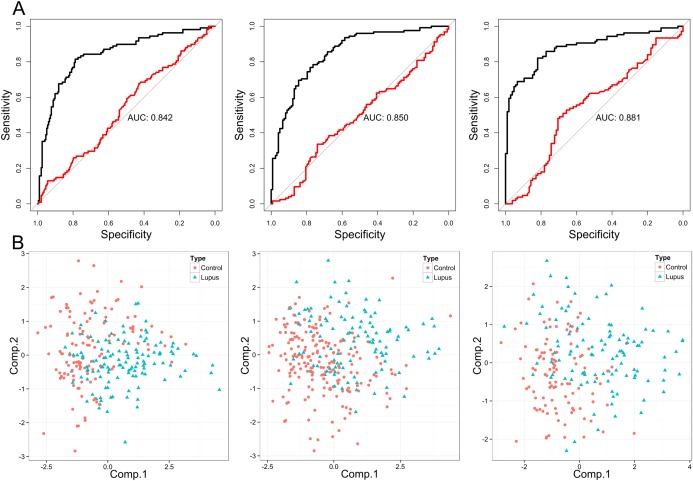
**A,** Receiver operating characteristic curves illustrating the performance of a regularized logistic regression model in predicting disease status of patients with systemic lupus erythematosus (SLE) and healthy controls in the African Caribbean (left), Latin American (middle), and Han Chinese (right) cohorts. While models based only on age and sex did not show predictive power (red lines), addition of glycan traits increased predictive power of models (black lines). AUC = area under the curve. **B,** Principal components analysis plots showing differences in GP6, GP9, GP10, and GP14 glycans between SLE patients and healthy controls in the African Caribbean (left), Latin American (middle), and Han Chinese (right) cohorts. Comp. = component (see Figure 1 for other definitions).

Since the majority of glycan structures were strongly associated with disease status, we tried to build a glycan‐based discriminative model using regularized logistic regression. In the discriminative model, only 24 directly measured glycan traits were used as predictors. To evaluate the biomarker potential of glycans, the model was trained and validated on each SLE cohort separately. For each cohort, a 10–cross‐validation procedure was used to evaluate model performance. Predictions from each validation round were merged into 1 large validation set on which performance of the cohort‐specific model was assessed using ROC curve analysis. While a model based on age and sex did not show significant discriminative power (AUC for African Caribbean cohort 0.537, AUC for Latin American cohort 0.498, AUC for Han Chinese cohort 0.552), addition of glycan variables into the model increased its discriminative power considerably in all 3 cohorts (AUC for African Caribbean cohort 0.842, AUC for Latin American cohort 0.850, AUC for Han Chinese cohort 0.881) (Figure 3A).

Examination of the classification performance of each individual glycan using ROC curve analysis identified a similar set of glycans as potential biomarkers in all 3 cohorts (see Supplementary Figure 3, available on the *Arthritis & Rheumatology* web site at http://onlinelibrary.wiley.com/doi/10.1002/art.39273/abstract). Glycan structure FA2[3]G1 (GP9) had the highest predictive power in each analyzed population, and structure FA2B (GP6) was the second strongest predictor, while structures FA2G2 (GP14) and FA2[6]BG1 (GP10) were among the strongest predictors in all 3 populations. Differences in GP6, GP9, GP10, and GP14 between patients and controls were visualized using PCA (Figure 3B). Since an increasing number of different SLE symptoms present in an individual patient was associated with progressive changes in the IgG glycome, we also attempted to build a glycan‐based discriminative model for SLE severity. Patients were divided into one group with no symptoms or 1 symptom and another group with 2–4 different symptoms. Regularized logistic regression based on IgG glycans enabled significantly improved classification compared to the basic model based on age and sex (AUC 0.68, *P* = 1 × 10^−7^).

## DISCUSSION

By analyzing IgG glycosylation in 3 independent cohorts of SLE patients, we revealed extensive and highly statistically significant changes in glycome composition. Interestingly, despite known differences in SLE manifestation in different ethnic groups, we observed very similar changes in IgG glycome composition in SLE patients of African Caribbean, Han Chinese, and Latin American Mestizo ethnicity. One of the prominently changed features of IgG in SLE was the extent of galactosylation. Since the initial discovery of decreased IgG galactosylation in rheumatoid arthritis (RA) in 1985, the same type of change in IgG glycosylation was reported in a number of different autoimmune and inflammatory diseases 23, and it was also associated with chronological and biologic age 22. Therefore, the decrease in galactosylation is clearly not disease‐specific but is instead a general phenomenon associated with decreased immunosuppressive potential of circulating IgG. However, the key unresolved question is whether this is a consequence of a disease or an individual variation that is a predisposition for a disease.

Our recent large population study of IgG glycosylation revealed very large interindividual variability in IgG glycome composition 21. The differences observed in the general population were of the same magnitude as (if not larger than) the differences that were previously reported in different diseases. Despite the absence of a direct genetic template, the heritability of individual glycans was very high (up to 80%) 21, 30, indicating that low IgG galactosylation could partly be a genetically predetermined predisposition. This hypothesis is further supported by the fact that in RA the decrease in IgG galactosylation was observed up to several years before the onset of the disease 31. However, galactosylation of IgG is also dynamic and can change quite rapidly in acute inflammation 32; thus, both genetic and environmental factors strongly affect IgG galactosylation. This is also supported by the fact that glycome composition is associated with both genetic polymorphisms and epigenetic modification on multiple genetic loci 8, 30. One genetic locus (*HLA–DQA2*) associated with IgG galactosylation 8 is also associated with SLE with genome‐wide significance 9, while a suggestive association with SLE was reported for another (*BACH2*) 10. Our results indicate that effects of decreased IgG galactosylation on inflammatory potential of IgG may be one of the molecular mechanisms that could explain the association between these genetic loci and SLE and/or autoimmunity.

In mice, Fc galactosylation has been shown to be crucial for antiinflammatory activity of antigen‐specific IgG1 immune complexes by promoting association of the inhibitory receptor FcγRIIb and the lectin‐like receptor dectin 1. This results in suppression of C5a receptor function, one of the important proinflammatory properties of complement. The complement system has a dual role in SLE, at the same time mediating pathogenesis and preventing the disease. Genetic and acquired deficiencies of components of the early part of the classical complement cascade (namely, C1q and C4, but also C2 and, rarely, C3) are associated with SLE 33, showing that the classical pathway of complement activation appears to protect against the development of SLE 34.

On the other hand, agalactosylated IgG (present at an increased level in all 3 populations tested) has lower affinity for C1q binding 35 but can bind serum mannose‐binding lectin (MBL) via terminally exposed GlcNAc and thus activate the lectin complement pathway 3, participating mechanistically in SLE pathogenesis. It is known that agalactosylated IgG binds to MBL and contributes to chronic inflammation in RA 36. The role of MBL and the lectin complement pathway in SLE pathogenesis is further supported by the increased serum MBL level found in SLE patients in various populations, leading to enhanced complement activation and tissue damage 37. Therefore, the high level of agalactosylated IgG in our patients probably contributes to SLE pathogenesis on 2 levels: 1) activating the lectin complement pathway (leading to tissue damage) via increased binding to MBL, and 2) suppressing the classical complement pathway (leading to impaired self‐antigen clearance) via decreased binding to C1q.

In contrast to changes in galactosylation, significant changes in sialylation, core fucose, and bisecting GlcNAc seem to be more specific for SLE. They were not previously reported to occur to this extent in other diseases, but analytic methods used until a few years ago were not able to reliably measure these IgG features; thus, these changes may have been overlooked in previous studies. The cross‐sectional nature of our study does not allow us to speculate about causality, but the increased potential of nonfucosylated IgG to activate ADCC has been clearly demonstrated in multiple studies 1. Genetic polymorphisms in Fc receptors are known risk factors for SLE 38; thus, it is reasonable to assume that differences in affinity of Fc receptors for differentially glycosylated IgG also have functional consequences in SLE.

In a very recent study of a small number of SLE patients (n = 15), Sjowall and colleagues showed increased binding of lectins recognizing fucose to complexed natural IgG in patients with active disease 39. This contradicts our observation that IgG fucosylation is decreased in SLE patients. However, Sjowall et al used an indirect lectin assay to analyze complexed and not isolated IgG, and, as they also suggested in their report, the target for the observed binding of *Aleuria aurantia* lectin may not be IgG itself but some other component of the complexed IgG.

The method that we used (UPLC analysis of released glycans) cannot differentiate glycans released from the Fab and the Fc portions of IgG. Glycans from Fab and Fc are known to be different, with Fab glycans having less core fucose and more galactose, sialic acid, and bisecting GlcNAc 40. Only ∼15% of the total IgG glycome originates from the Fab portion of IgG; thus, the observed differences between patients and controls presumably originate from the Fc glycans. Using this analytic method, we cannot exclude the possibility that the decrease of core fucose in SLE is driven by an increase in the extent of Fab glycosylation (and consequent decrease in the proportion of core fucose in the total glycome). However, in that case we would also expect to see an increase in sialylation, while we observed a decrease instead. Therefore, the observed differences most probably originate from altered regulation of Fc glycosylation in SLE patients.

Decreased IgG sialylation significantly reduces antiinflammatory activity of circulating IgG 41. IVIG is currently not used extensively to treat SLE, but our results indicate that decreased antiinflammatory function of circulating IgG could be one of the molecular mechanisms underlying SLE pathology. The usual dose of IVIG for suppression of autoimmune diseases is 1–3 gm/kg/month. However, with a plasma IgG concentration of up to 15 gm/liter and ∼50% of that in extracellular fluids, at any given moment the total IgG concentration in the body exceeds the maximal therapeutic dose used to treat different inflammatory and autoimmune diseases. IgG glycans have been shown to be crucial for efficacy of IVIG therapy 41; thus, in principle we are all receiving continuous “internal IVIG therapy” that is strongly affected by individual variations in IgG glycosylation. With the exception of immune deficiencies, in which IVIG is administered at lower doses, nearly all diseases in which IVIG is used as therapy at high doses are associated with an IgG glycome profile that is significantly more proinflammatory; thus, IVIG therapy could actually only be correcting this imbalance. Concordant with this hypothesis are results of a recent study that indicate that individual differences in IgG sialylation are predictive of the response to IVIG therapy in Kawasaki disease 42, an immune febrile vasculitis syndrome of early childhood. In addition, 2 minor alleles of the gene for FcγRIIb (−386C and −120A) conferring increased promoter activity and consequently an increased level of receptor expression are positively correlated with therapeutic response to IVIG in patients with Kawasaki disease 43. Interestingly, however, these alleles are also risk factors for SLE 38, which is not in accordance with the well‐established protective role of FcγRIIb against the development of self‐reactive response, as demonstrated in FcγRIIb‐deficient mice of certain strains that spontaneously develop autoimmune disorders 44. Although there are observations that indicate associations of several of its variants with SLE 38, the role of the inhibiting FcγRIIb receptor in SLE has never been demonstrated in non‐Asian populations. It may act as a modifier of autoimmune susceptibility, with a minor role in tolerance maintenance in the efferent phase (autoantibody production), but with important roles in regulation of downstream antibody effector pathways, such as immune complex clearance and enhanced myeloid effector cell responses.

Genetic loci associated with IgG glycome composition have pleiotropic effects on multiple inflammatory diseases, autoimmune diseases, and cancer 8, indicating that differences in IgG glycosylation and their functional consequences may affect balance of the immune system and contribute to the increased risk of development of various diseases. SLE patients have also been reported to express low levels of Ikaros family zinc‐finger protein 1 (IKZF1) in peripheral blood 9. IKZF1 is important for regulation of self tolerance through B cell receptor signaling, but the molecular mechanism is not known. *IKZF1* was a major hit for core fucose in the IgG glycome GWAS 8; thus, altered IgG core fucosylation is a plausible mechanism for distorted interactions of IgG with Fc receptors in SLE. At the same time, *HLA–DQ2A/B* and *BACH2* genetic loci are associated with IgG galactosylation 8, and thus their association with SLE could reflect subdued immunosuppressive activity of IgG in SLE, which suggests that changes in IgG glycosylation could be one molecular mechanism by which these genes affect SLE. Variation in IgG glycosylation is associated with disease risk and severity of symptoms, and this phenomenon should be explored further to understand its importance in a personalized approach to treating SLE patients. It would be particularly interesting to evaluate longitudinal dynamics of the IgG glycome within an individual patient at different stages of the disease and/or receiving therapy with different immunomodulatory drugs.

## AUTHOR CONTRIBUTIONS

All authors were involved in drafting the article or revising it critically for important intellectual content, and all authors approved the final version to be published. Drs. Alarcon‐Riquelme, Molokhia, Wang, and Lauc had full access to all of the data in the study and take responsibility for the integrity of the data and the accuracy of the data analysis.

### Study conception and design

Gornik, Alarcon‐Riquelme, W. Wang, Lauc.

### Acquisition of data

Krištić, Gudelj, Keser, Pezer, Pučić‐Baković, Štambuk, Pavić, Y. Wang, Zhou, Cui, Song, Zeng, Guo, McKeigue, Spector, Alarcon‐Riquelme, Molokhia, W. Wang.

### Analysis and interpretation of data

Vučković, Teruel, Trbojević‐Akmačić, Barrios, Menni, Zeng, Guo, Pons‐Estel, McKeigue, Patrick, Gornik, Harjaček, Alarcon‐Riquelme, Molokhia, W. Wang, Lauc.

## ADDITIONAL DISCLOSURES

Authors Vučković, Krištić, Gudelj, Pezer, Pučić‐Baković, Štambuk, and Trbojević‐Akmačić are employees of Genos Ltd. Author Lauc is the founder and chief executive officer of Genos Ltd.

## Supporting information


**Supplementary Table 1.** Clinical characteristics of patients with lupus and control subjects.
**Supplementary Table 2.** Odds ratios (OR), 95% confidence intervals (95% CI) and *P* values for the associations of glycan traits with disease status adjusted for age and gender.
**Supplementary Table 3.** Clinical features associated with glycan traits in SLE.
**Supplementary Figure 1. Functional relevance of IgG glycosylation**. Glycans play an important functional role on all immunoglobulins. When attached to the conserved Asn297 in IgG heavy chain they significantly participate in the structure and the overall conformation of IgG conserved region. Deglycosylated IgG can not activate effector functions, while the attachment of different glycans to this site promotes binding to different Fc receptors and in this way modulate effector functions (45). Between 15% and 20% of IgG also bears glycans on the variable region of IgG, but functional relevance of these glycans is not known.
**Supplementary Figure 2. Association of glycosylation changes with SLE related clinical features in Afro‐ Caribbean (n = 110) and Latino American (n = 261) cohorts.** Changes in glycosylation were more pronounced in patients with positive ANA test, pericarditis, proteinuria or in patients with disease duration of over 8 years. While bisecting GlcNAc was increased in patients with severe symptoms, sialylation, galactosylation and fucosylation were in most cases decreased.
* G0n = Proportion of agalactosylated structures in neutral glycans; G2n = Proportion of digalactosylated structures in neutral glycans; Fn total = Proportion of fucosylated structures in neutral glycans; S1 total = Proportion of monosialylated structures in total IgG glycans; FBn = Proportion of fucosylated (with bisecting GlcNAc) structures in total neutral IgG glycans.
**Supplementary Figure 3. Variable importance in glycan based SLE discriminative models.** Predictive power of each individual glycan trait was evaluated by ROC curve analysis on (A) Afro‐Caribbean, (B) Latin American and (C) Chinese cohort.
