# Supplementary material for: Association of Systemic Lupus Erythematosus With Decreased Immunosuppressive Potential of the IgG Glycome
Source: Arthritis Rheumatol. 2015 Oct 28;67(11):2978–89. doi: 10.1002/art.39273 (PMC4626261; doi:10.1002/art.39273)
Supplement: Supplementary file 1 — Supplementary Table 1. Clinical characteristics of patients with lupus and control subjects. Supplementary Table 2. Odds ratios (OR), 95% confidence intervals (95% CI) and P values for the associations of glycan traits with disease status adjusted for age and gender. Supplementary Table 3. Clinical features associated with glycan traits in SLE. Supplementary Figure 1. Functional relevance of IgG glycosylation. Glycans play an important functional role on all immunoglobulins. When attached to the conserved Asn297 in IgG heavy chain they significantly participate in the structure and the overall conformation of IgG conserved region. Deglycosylated IgG can not activate effector functions, while the attachment of different glycans to this site promotes binding to different Fc receptors and in this way modulate effector functions (45). Between 15% and 20% of IgG also bears glycans on the variable region of IgG, but functional relevance of these glycans is not known. Supplementary Figure 2. Association of glycosylation changes with SLE related clinical features in Afro‐ Caribbean (n = 110) and Latino American (n = 261) cohorts. Changes in glycosylation were more pronounced in patients with positive ANA test, pericarditis, proteinuria or in patients with disease duration of over 8 years. While bisecting GlcNAc was increased in patients with severe symptoms, sialylation, galactosylation and fucosylation were in most cases decreased. * G0n = Proportion of agalactosylated structures in neutral glycans; G2n = Proportion of digalactosylated structures in neutral glycans; Fn total = Proportion of fucosylated structures in neutral glycans; S1 total = Proportion of monosialylated structures in total IgG glycans; FBn = Proportion of fucosylated (with bisecting GlcNAc) structures in total neutral IgG glycans. Supplementary Figure 3. Variable importance in glycan based SLE discriminative models. Predictive power of each individual glycan trait was evaluated by ROC curve analysis on ( [file ART-67-2978-s001.docx]

**Systemic lupus erythematosus associates with the decreased immunosuppressive potential of the IgG**

**glycome**

Frano Vučković1,*, Jasminka Krištić1,*, Maria Teruel Artacho2, Toma Keser3, Ivan Gudelj1, Marija Pezer1, Maja Pučić-Baković1,, Jerko Štambuk1, Irena

Trbojević-Akmačić1, Clara Barrios4, Cristina Menni4, Youxin Wang5, Yong Zhou6, Liufu Cui7, Haicheng Song8, Qiang Zeng9, Xiuhua Guo5, Bernardo Pons-

Estel10, Paul McKeigue11, Alan Patrick12, Tamara Pavic3, Olga Gornik3, Tim D. Spector4, Miroslav Harjaček13, Marta Alarcon-Riquelme2,14,**, Mariam

Molokhia15,**, Wei Wang5,16,**, and Gordan Lauc1,3,**

**SUPPLEMENTARY INFORMATION**

**Supplementary Table 1.** Clinical characteristics of patients with lupus and control subjects.

|  | **Afro-Caribbean** | | **Latin American** | | **Chinese** | |
| --- | --- | --- | --- | --- | --- | --- |
|  | **Controls**  **(N=194)** | **Patients**  **(N=110)** | **Controls**  **(N=247)** | **Patients**  **(N=261)** | **Controls**  **(N=105)** | **Patients**  **(N=106)** |
| **Age (yr)** | 45.5 (39.3-57.0) | 47.0 (41.0-54.0) | 38 (29-49) | 39 (29-49) | 36.7 (28-43) | 36.6 (28-45.5) |
| **Female sex (%)** | 92 | 91 | 91 | 90 | 85 | 93 |
| **Disease Duration**  **(years)** | 0 | 13.0 (8.0-19.0) | 0 | 7 (3-14) | 0 | 4 (1-7) |
| **ANA test (%)** | 0 | 69 | 0 | 76 | 0 | N/A |
| **Proteinuria (%)** | 0 | 30 | 0 | 45 | 0 | N/A |
| **Pericarditis (%)** | 0 | 21 | 0 | 18 | 0 | N/A |

**Supplementary Table 2.** Odds ratios (OR), 95% confidence intervals (95% CI) and p values for the associations of glycan traits with disease status adjusted for

age and gender.

|  | **Afro-Caribbean** | | | **Latin American** | | | **Chinese** | | | **Meta** | | |
| --- | --- | --- | --- | --- | --- | --- | --- | --- | --- | --- | --- | --- |
| **Glycan** | **effect** | **conf_int** | **p.val** | **effect** | **conf_int** | **p.val** | **effect** | **conf_int** | **p.val** | **effect** | **conf_int** | **p.adj** |
| GP1 | 1,65 | (1,27 - 2,15) | 1,74E-04 | 1,93 | (1,56 - 2,37) | 1,95E-10 | 2,63 | (1,82 - 3,82) | 3,32E-08 | 1,93 | (1,66 - 2,24) | 1,88E-17 |
| GP2 | 1,56 | (1,20 - 2,03) | 1,22E-03 | 2,38 | (1,87 - 3,03) | 5,30E-14 | 2,44 | (1,70 - 3,49) | 1,69E-07 | 2,05 | (1,75 - 2,40) | 5,40E-18 |
| GP4 | 1,70 | (1,29 - 2,24) | 1,52E-04 | 1,87 | (1,50 - 2,33) | 1,10E-08 | 1,83 | (1,32 - 2,53) | 2,48E-04 | 1,81 | (1,55 - 2,11) | 4,33E-14 |
| GP5 | 0,97 | (0,76 - 1,24) | 8,32E-01 | 1,48 | (1,22 - 1,79) | 6,39E-05 | 1,74 | (1,27 - 2,38) | 4,47E-04 | 1,34 | (1,17 - 1,53) | 3,00E-05 |
| GP6 | 2,42 | (1,77 - 3,29) | 3,40E-09 | 3,16 | (2,40 - 4,17) | 1,09E-19 | 4,14 | (2,65 - 6,48) | 9,32E-14 | 3,00 | (2,49 - 3,62) | 7,38E-30 |
| GP7 | 1,18 | (0,93 - 1,50) | 2,01E-01 | 1,74 | (1,41 - 2,14) | 1,03E-07 | 1,94 | (1,40 - 2,68) | 3,77E-05 | 1,56 | (1,35 - 1,79) | 1,47E-09 |
| GP8 | 0,81 | (0,64 - 1,04) | 1,12E-01 | 0,97 | (0,81 - 1,16) | 7,69E-01 | 0,65 | (0,48 - 0,88) | 6,47E-03 | 0,86 | (0,75 - 0,97) | 2,40E-02 |
| GP9 | 0,20 | (0,13 - 0,30) | 2,15E-21 | 0,31 | (0,24 - 0,40) | 1,26E-24 | 0,23 | (0,15 - 0,36) | 2,82E-14 | 0,27 | (0,22 - 0,33) | 2,08E-39 |
| GP10 | 1,68 | (1,29 - 2,18) | 1,21E-04 | 1,95 | (1,59 - 2,41) | 6,44E-11 | 3,24 | (2,17 - 4,84) | 5,52E-11 | 2,00 | (1,72 - 2,32) | 1,02E-18 |
| GP11 | 0,85 | (0,67 - 1,08) | 2,03E-01 | 1,14 | (0,94 - 1,37) | 1,96E-01 | 1,56 | (1,14 - 2,13) | 6,34E-03 | 1,10 | (0,96 - 1,26) | 1,80E-01 |
| GP12 | 1,05 | (0,82 - 1,36) | 7,24E-01 | 1,19 | (0,99 - 1,42) | 7,79E-02 | 1,45 | (1,07 - 1,97) | 1,90E-02 | 1,19 | (1,05 - 1,36) | 1,11E-02 |
| GP13 | 0,74 | (0,57 - 0,96) | 2,61E-02 | 1,03 | (0,86 - 1,23) | 7,94E-01 | 1,56 | (1,15 - 2,10) | 4,76E-03 | 1,02 | (0,89 - 1,16) | 7,89E-01 |
| GP14 | 0,41 | (0,30 - 0,57) | 1,28E-08 | 0,34 | (0,26 - 0,45) | 3,86E-18 | 0,36 | (0,25 - 0,53) | 1,36E-08 | 0,37 | (0,31 - 0,44) | 9,74E-27 |
| GP15 | 1,08 | (0,84 - 1,38) | 5,90E-01 | 0,84 | (0,69 - 1,02) | 8,85E-02 | 1,14 | (0,85 - 1,52) | 4,18E-01 | 0,97 | (0,84 - 1,11) | 6,50E-01 |
| GP16 | 0,77 | (0,60 - 0,99) | 4,76E-02 | 0,53 | (0,43 - 0,65) | 5,23E-10 | 0,59 | (0,43 - 0,80) | 8,23E-04 | 0,61 | (0,53 - 0,71) | 1,63E-11 |
| GP17 | 0,84 | (0,66 - 1,07) | 1,73E-01 | 1,28 | (1,07 - 1,54) | 9,37E-03 | 2,12 | (1,51 - 2,98) | 7,18E-06 | 1,22 | (1,06 - 1,39) | 5,28E-03 |
| GP18 | 0,70 | (0,54 - 0,91) | 1,07E-02 | 0,44 | (0,34 - 0,56) | 1,77E-12 | 0,56 | (0,40 - 0,78) | 4,96E-04 | 0,55 | (0,47 - 0,64) | 1,23E-13 |
| GP19 | 1,07 | (0,84 - 1,36) | 6,53E-01 | 1,54 | (1,28 - 1,87) | 7,93E-06 | 1,19 | (0,90 - 1,58) | 2,59E-01 | 1,31 | (1,14 - 1,49) | 1,01E-04 |
| GP20 | 0,76 | (0,59 - 0,97) | 3,46E-02 | 0,71 | (0,59 - 0,85) | 3,25E-04 | 1,00 | (0,75 - 1,33) | 9,97E-01 | 0,78 | (0,68 - 0,89) | 2,26E-04 |
| GP21 | 0,80 | (0,62 - 1,02) | 8,31E-02 | 0,96 | (0,80 - 1,14) | 6,65E-01 | 0,97 | (0,73 - 1,29) | 8,69E-01 | 0,91 | (0,80 - 1,04) | 1,87E-01 |
| GP22 | 1,19 | (0,93 - 1,52) | 1,81E-01 | 1,72 | (1,42 - 2,10) | 3,60E-08 | 2,18 | (1,56 - 3,05) | 1,59E-06 | 1,59 | (1,38 - 1,83) | 1,11E-10 |
| GP23 | 0,68 | (0,53 - 0,87) | 3,05E-03 | 0,66 | (0,54 - 0,80) | 3,77E-05 | 0,34 | (0,24 - 0,50) | 1,27E-09 | 0,61 | (0,53 - 0,70) | 1,63E-11 |
| GP24 | 1,51 | (1,17 - 1,95) | 1,88E-03 | 1,81 | (1,48 - 2,20) | 2,20E-09 | 1,34 | (1,01 - 1,79) | 5,26E-02 | 1,60 | (1,40 - 1,84) | 3,29E-11 |
| FGS/(FG+FGS) | 1,04 | (0,82 - 1,33) | 7,76E-01 | 0,75 | (0,62 - 0,90) | 3,93E-03 | 0,99 | (0,74 - 1,33) | 9,45E-01 | 0,88 | (0,77 - 1,00) | 6,57E-02 |
| FBGS/(FBG+FBGS) | 1,03 | (0,81 - 1,31) | 8,13E-01 | 1,26 | (1,06 - 1,52) | 1,41E-02 | 0,73 | (0,54 - 0,97) | 3,75E-02 | 1,07 | (0,94 - 1,22) | 3,43E-01 |
| FGS/(F+FG+FGS) | 0,76 | (0,59 - 0,98) | 4,51E-02 | 0,55 | (0,44 - 0,68) | 4,74E-08 | 0,67 | (0,49 - 0,91) | 1,40E-02 | 0,64 | (0,55 - 0,74) | 4,14E-09 |
| FBGS/(FB+FBG+FBGS) | 0,83 | (0,65 - 1,06) | 1,49E-01 | 0,96 | (0,80 - 1,16) | 7,39E-01 | 0,54 | (0,39 - 0,74) | 9,43E-05 | 0,83 | (0,73 - 0,95) | 8,30E-03 |
| FG1S1/(FG1+FG1S1) | 1,21 | (0,95 - 1,54) | 1,46E-01 | 0,76 | (0,63 - 0,91) | 4,76E-03 | 1,07 | (0,81 - 1,42) | 6,65E-01 | 0,94 | (0,82 - 1,06) | 3,43E-01 |
| FG2S1/(FG2+FG2S1+FG2S2) | 1,93 | (1,47 - 2,53) | 1,02E-06 | 1,31 | (1,09 - 1,57) | 4,81E-03 | 1,96 | (1,42 - 2,70) | 2,53E-05 | 1,55 | (1,36 - 1,78) | 4,53E-10 |
| FG2S2/(FG2+FG2S1+FG2S2) | 1,05 | (0,81 - 1,35) | 7,69E-01 | 1,32 | (1,10 - 1,60) | 4,83E-03 | 0,89 | (0,67 - 1,19) | 4,85E-01 | 1,14 | (0,99 - 1,30) | 7,21E-02 |
| FBG2S1/(FBG2+FBG2S1+FBG2S2) | 0,64 | (0,50 - 0,83) | 8,64E-04 | 1,20 | (1,00 - 1,44) | 6,55E-02 | 0,90 | (0,68 - 1,21) | 5,46E-01 | 0,96 | (0,84 - 1,09) | 5,41E-01 |
| FBG2S2/(FBG2+FBG2S1+FBG2S2) | 1,63 | (1,26 - 2,13) | 2,71E-04 | 1,86 | (1,52 - 2,28) | 6,40E-10 | 1,24 | (0,93 - 1,66) | 1,62E-01 | 1,63 | (1,42 - 1,88) | 1,63E-11 |
| FtotalS1/FtotalS2 | 0,70 | (0,53 - 0,92) | 1,18E-02 | 0,47 | (0,37 - 0,58) | 7,13E-13 | 0,89 | (0,67 - 1,19) | 4,80E-01 | 0,62 | (0,54 - 0,72) | 4,53E-10 |
| FS1/FS2 | 1,23 | (0,95 - 1,58) | 1,35E-01 | 0,87 | (0,72 - 1,04) | 1,45E-01 | 1,65 | (1,21 - 2,24) | 1,42E-03 | 1,08 | (0,94 - 1,23) | 3,08E-01 |
| FBS1/FBS2 | 0,58 | (0,45 - 0,75) | 4,20E-05 | 0,67 | (0,56 - 0,81) | 3,15E-05 | 0,74 | (0,55 - 0,99) | 5,23E-02 | 0,66 | (0,57 - 0,75) | 2,16E-09 |
| FBStotal/FStotal | 2,29 | (1,67 - 3,13) | 4,74E-08 | 4,15 | (3,08 - 5,60) | 1,19E-28 | 1,99 | (1,42 - 2,78) | 3,41E-05 | 2,74 | (2,28 - 3,28) | 7,87E-27 |
| FBS1/FS1 | 1,52 | (1,16 - 1,99) | 3,13E-03 | 2,93 | (2,27 - 3,79) | 9,51E-20 | 1,71 | (1,25 - 2,36) | 8,77E-04 | 2,03 | (1,73 - 2,38) | 2,23E-17 |
| FBS1/(FS1+FBS1) | 1,52 | (1,16 - 1,99) | 3,13E-03 | 2,93 | (2,27 - 3,79) | 9,51E-20 | 1,71 | (1,25 - 2,36) | 8,77E-04 | 2,03 | (1,73 - 2,38) | 2,23E-17 |
| FBS2/FS2 | 3,51 | (2,47 - 4,99) | 1,09E-15 | 4,42 | (3,29 - 5,95) | 3,36E-32 | 4,08 | (2,61 - 6,36) | 1,68E-13 | 4,03 | (3,29 - 4,93) | 2,39E-40 |
| FBS2/(FS2+FBS2) | 3,51 | (2,47 - 4,99) | 1,09E-15 | 4,42 | (3,29 - 5,95) | 3,36E-32 | 4,08 | (2,61 - 6,36) | 1,68E-13 | 4,03 | (3,29 - 4,93) | 2,39E-40 |
| S total | 0,79 | (0,62 - 1,01) | 7,68E-02 | 0,65 | (0,53 - 0,80) | 3,92E-05 | 0,63 | (0,46 - 0,86) | 4,66E-03 | 0,69 | (0,60 - 0,79) | 3,13E-07 |
| S1 total | 0,70 | (0,54 - 0,91) | 9,29E-03 | 0,50 | (0,40 - 0,63) | 8,85E-10 | 0,62 | (0,45 - 0,86) | 4,49E-03 | 0,59 | (0,51 - 0,69) | 1,63E-11 |
| S2 total | 0,95 | (0,75 - 1,21) | 7,11E-01 | 1,13 | (0,95 - 1,36) | 1,91E-01 | 0,78 | (0,58 - 1,04) | 1,01E-01 | 1,00 | (0,88 - 1,14) | 9,99E-01 |
| GP1n | 1,64 | (1,26 - 2,13) | 2,05E-04 | 1,89 | (1,54 - 2,32) | 4,30E-10 | 2,65 | (1,83 - 3,85) | 2,42E-08 | 1,91 | (1,64 - 2,21) | 3,57E-17 |
| GP2n | 1,55 | (1,19 - 2,01) | 1,48E-03 | 2,34 | (1,85 - 2,98) | 8,23E-14 | 2,44 | (1,70 - 3,49) | 1,54E-07 | 2,03 | (1,73 - 2,38) | 7,04E-18 |
| GP4n | 1,83 | (1,38 - 2,43) | 2,03E-05 | 1,90 | (1,52 - 2,36) | 5,11E-09 | 1,82 | (1,31 - 2,53) | 2,54E-04 | 1,86 | (1,60 - 2,17) | 3,82E-15 |
| GP5n | 0,92 | (0,72 - 1,18) | 5,61E-01 | 1,36 | (1,13 - 1,63) | 1,39E-03 | 1,65 | (1,22 - 2,24) | 1,32E-03 | 1,26 | (1,10 - 1,44) | 8,60E-04 |
| GP6n | 2,59 | (1,89 - 3,56) | 3,21E-10 | 3,32 | (2,50 - 4,39) | 6,23E-21 | 4,62 | (2,89 - 7,39) | 4,88E-15 | 3,20 | (2,64 - 3,88) | 1,21E-31 |
| GP7n | 1,16 | (0,91 - 1,48) | 2,47E-01 | 1,66 | (1,36 - 2,03) | 6,53E-07 | 1,93 | (1,40 - 2,67) | 3,92E-05 | 1,52 | (1,32 - 1,74) | 7,24E-09 |
| GP8n | 0,71 | (0,55 - 0,91) | 9,99E-03 | 0,80 | (0,66 - 0,98) | 3,41E-02 | 0,55 | (0,40 - 0,76) | 1,72E-04 | 0,72 | (0,63 - 0,83) | 4,58E-06 |
| GP9n | 0,18 | (0,12 - 0,28) | 2,35E-23 | 0,26 | (0,20 - 0,34) | 8,94E-30 | 0,18 | (0,11 - 0,30) | 4,73E-17 | 0,22 | (0,18 - 0,28) | 2,36E-43 |
| GP10n | 1,56 | (1,20 - 2,02) | 8,64E-04 | 1,73 | (1,42 - 2,11) | 3,93E-08 | 2,87 | (1,97 - 4,19) | 1,13E-09 | 1,80 | (1,56 - 2,08) | 3,82E-15 |
| GP11n | 0,78 | (0,61 - 1,00) | 6,44E-02 | 1,04 | (0,87 - 1,25) | 7,20E-01 | 1,46 | (1,08 - 1,98) | 1,71E-02 | 1,02 | (0,90 - 1,17) | 7,62E-01 |
| GP12n | 1,02 | (0,79 - 1,32) | 8,69E-01 | 1,13 | (0,95 - 1,36) | 1,95E-01 | 1,41 | (1,04 - 1,91) | 3,41E-02 | 1,15 | (1,01 - 1,31) | 4,90E-02 |
| GP13n | 0,72 | (0,55 - 0,93) | 1,37E-02 | 0,96 | (0,80 - 1,15) | 6,87E-01 | 1,41 | (1,05 - 1,90) | 2,74E-02 | 0,96 | (0,84 - 1,09) | 5,54E-01 |
| GP14n | 0,45 | (0,33 - 0,62) | 1,62E-07 | 0,36 | (0,28 - 0,47) | 6,15E-17 | 0,39 | (0,26 - 0,56) | 6,04E-08 | 0,39 | (0,33 - 0,47) | 1,33E-24 |
| GP15n | 0,98 | (0,77 - 1,26) | 9,02E-01 | 0,79 | (0,65 - 0,96) | 2,54E-02 | 1,04 | (0,78 - 1,39) | 8,10E-01 | 0,90 | (0,78 - 1,03) | 1,41E-01 |
| G0n | 2,17 | (1,61 - 2,93) | 1,51E-07 | 2,33 | (1,83 - 2,96) | 2,36E-13 | 2,47 | (1,71 - 3,55) | 1,57E-07 | 2,31 | (1,95 - 2,72) | 3,14E-22 |
| G1n | 0,50 | (0,38 - 0,66) | 3,24E-07 | 0,64 | (0,52 - 0,78) | 7,93E-06 | 0,44 | (0,31 - 0,62) | 7,72E-07 | 0,56 | (0,48 - 0,64) | 8,42E-15 |
| G2n | 0,51 | (0,37 - 0,68) | 5,82E-06 | 0,43 | (0,33 - 0,54) | 2,38E-13 | 0,48 | (0,34 - 0,69) | 2,20E-05 | 0,46 | (0,39 - 0,55) | 2,67E-19 |
| Fn total | 0,82 | (0,65 - 1,05) | 1,40E-01 | 0,65 | (0,54 - 0,79) | 1,07E-05 | 0,51 | (0,37 - 0,71) | 3,77E-05 | 0,67 | (0,59 - 0,77) | 1,62E-08 |
| FG0n total/G0n | 0,82 | (0,64 - 1,05) | 1,35E-01 | 0,60 | (0,49 - 0,74) | 5,13E-07 | 0,51 | (0,37 - 0,71) | 3,95E-05 | 0,65 | (0,56 - 0,74) | 1,73E-09 |
| FG1n total/G1n | 0,79 | (0,62 - 1,00) | 6,53E-02 | 0,55 | (0,45 - 0,68) | 1,53E-08 | 0,47 | (0,34 - 0,66) | 3,89E-06 | 0,61 | (0,53 - 0,70) | 1,63E-11 |
| FG2n total /G2n | 0,68 | (0,53 - 0,87) | 3,05E-03 | 0,45 | (0,36 - 0,56) | 9,32E-14 | 0,40 | (0,28 - 0,57) | 4,07E-08 | 0,51 | (0,44 - 0,59) | 7,99E-18 |
| Fn | 0,49 | (0,37 - 0,65) | 2,75E-07 | 0,37 | (0,29 - 0,47) | 1,08E-18 | 0,18 | (0,11 - 0,30) | 1,98E-17 | 0,38 | (0,32 - 0,45) | 4,61E-28 |
| FG0n/G0n | 0,70 | (0,55 - 0,91) | 7,75E-03 | 0,60 | (0,49 - 0,74) | 4,82E-07 | 0,36 | (0,24 - 0,52) | 2,64E-09 | 0,59 | (0,51 - 0,68) | 8,42E-13 |
| FG1n/G1n | 0,47 | (0,35 - 0,62) | 4,39E-08 | 0,40 | (0,32 - 0,50) | 3,34E-17 | 0,20 | (0,12 - 0,32) | 1,26E-16 | 0,39 | (0,33 - 0,46) | 4,61E-28 |
| FG2n/G2n | 0,46 | (0,35 - 0,61) | 2,53E-08 | 0,28 | (0,22 - 0,37) | 1,25E-24 | 0,22 | (0,14 - 0,36) | 5,67E-15 | 0,33 | (0,28 - 0,40) | 1,62E-31 |
| FBn | 2,15 | (1,61 - 2,87) | 4,95E-08 | 2,65 | (2,08 - 3,36) | 6,84E-18 | 5,09 | (3,15 - 8,23) | 1,48E-16 | 2,67 | (2,25 - 3,17) | 2,06E-28 |
| FBG0n/G0n | 1,38 | (1,08 - 1,77) | 1,42E-02 | 1,50 | (1,24 - 1,82) | 4,75E-05 | 2,32 | (1,64 - 3,28) | 3,78E-07 | 1,57 | (1,36 - 1,80) | 4,71E-10 |
| FBG1n/G1n | 2,06 | (1,55 - 2,73) | 1,77E-07 | 2,27 | (1,82 - 2,83) | 1,44E-14 | 3,85 | (2,52 - 5,88) | 2,12E-13 | 2,37 | (2,02 - 2,78) | 3,00E-25 |
| FBG2n/G2n | 2,72 | (1,97 - 3,75) | 4,22E-11 | 2,85 | (2,22 - 3,65) | 9,00E-20 | 3,96 | (2,56 - 6,11) | 1,89E-13 | 2,97 | (2,48 - 3,55) | 1,06E-31 |
| FBn/Fn | 2,16 | (1,62 - 2,88) | 4,39E-08 | 2,71 | (2,13 - 3,45) | 1,72E-18 | 5,38 | (3,28 - 8,81) | 3,34E-17 | 2,72 | (2,28 - 3,23) | 8,21E-29 |
| FBn/Fn total | 2,16 | (1,62 - 2,88) | 4,39E-08 | 2,71 | (2,13 - 3,45) | 1,72E-18 | 5,38 | (3,28 - 8,81) | 3,34E-17 | 2,72 | (2,28 - 3,23) | 8,21E-29 |
| Fn/(Bn + FBn) | 0,47 | (0,35 - 0,63) | 7,80E-08 | 0,37 | (0,29 - 0,47) | 3,11E-18 | 0,18 | (0,11 - 0,30) | 2,65E-17 | 0,37 | (0,31 - 0,44) | 2,06E-28 |
| Bn/(Fn + FBn) | 0,73 | (0,56 - 0,94) | 1,82E-02 | 0,97 | (0,82 - 1,16) | 8,01E-01 | 1,48 | (1,09 - 1,99) | 1,35E-02 | 0,98 | (0,86 - 1,11) | 7,48E-01 |
| FBG2n/FG2n | 2,82 | (2,04 - 3,89) | 7,50E-12 | 3,24 | (2,49 - 4,21) | 5,98E-23 | 4,90 | (3,06 - 7,86) | 3,83E-16 | 3,30 | (2,73 - 3,98) | 1,17E-34 |
| FBG2n /(FG2n + FBG2n ) | 2,82 | (2,04 - 3,89) | 7,50E-12 | 3,24 | (2,49 - 4,21) | 5,98E-23 | 4,90 | (3,06 - 7,86) | 3,83E-16 | 3,30 | (2,73 - 3,98) | 1,17E-34 |
| FG2n/(BG2n + FBG2n) | 0,37 | (0,27 - 0,51) | 5,18E-11 | 0,31 | (0,24 - 0,41) | 2,31E-22 | 0,18 | (0,11 - 0,30) | 2,65E-17 | 0,31 | (0,26 - 0,37) | 1,19E-33 |
| BG2n/(FG2n + FBG2n) | 1,30 | (1,02 - 1,66) | 4,47E-02 | 2,18 | (1,73 - 2,75) | 2,94E-12 | 4,47 | (2,82 - 7,08) | 2,04E-14 | 1,92 | (1,64 - 2,25) | 1,50E-15 |

**Supplementary Table 3.** Clinical features associated with glycan traits in SLE.

| **Glycan** | **Trait** | Latin American | | | Afro-Caribbean | | | Meta | | |
| --- | --- | --- | --- | --- | --- | --- | --- | --- | --- | --- |
|  |  | **effect** | **SE** | **p.val** | **effect** | **SE** | **p.val** | **effect** | **SE** | **p.adj** |
| FBS2/(FS2+FBS2) | ana | 0,747 | 0,134 | 3,24E-08 | 0,449 | 0,227 | 4,06E-02 | 0,670 | 0,115 | 2,53E-06 |
| FBS2/FS2 | ana | 0,747 | 0,134 | 3,24E-08 | 0,449 | 0,227 | 4,06E-02 | 0,670 | 0,115 | 2,53E-06 |
| GP9n | ana | -0,666 | 0,138 | 1,65E-06 | -0,600 | 0,225 | 6,18E-03 | -0,648 | 0,118 | 1,13E-05 |
| GP9 | ana | -0,616 | 0,141 | 1,31E-05 | -0,532 | 0,226 | 1,49E-02 | -0,592 | 0,120 | 1,65E-04 |
| FBG2n /(FG2n + FBG2n ) | ana | 0,663 | 0,134 | 8,52E-07 | 0,091 | 0,224 | 6,71E-01 | 0,513 | 0,115 | 1,00E-03 |
| FBG2n/FG2n | ana | 0,663 | 0,134 | 8,52E-07 | 0,091 | 0,224 | 6,71E-01 | 0,513 | 0,115 | 1,00E-03 |
| FBn/Fn | ana | 0,576 | 0,134 | 1,74E-05 | 0,302 | 0,231 | 1,73E-01 | 0,507 | 0,116 | 1,00E-03 |
| FBn/Fn total | ana | 0,576 | 0,134 | 1,74E-05 | 0,302 | 0,231 | 1,73E-01 | 0,507 | 0,116 | 1,00E-03 |
| FG2n/(BG2n + FBG2n) | ana | -0,637 | 0,133 | 1,92E-06 | -0,101 | 0,225 | 6,37E-01 | -0,498 | 0,115 | 1,00E-03 |
| Fn/(Bn + FBn) | ana | -0,569 | 0,134 | 2,21E-05 | -0,307 | 0,231 | 1,66E-01 | -0,503 | 0,116 | 1,00E-03 |
| FBG2n/G2n | ana | 0,654 | 0,136 | 1,68E-06 | 0,096 | 0,224 | 6,52E-01 | 0,503 | 0,116 | 1,00E-03 |
| FBn | ana | 0,587 | 0,134 | 1,21E-05 | 0,250 | 0,232 | 2,59E-01 | 0,503 | 0,116 | 1,00E-03 |
| FG1n/G1n | ana | -0,571 | 0,136 | 2,59E-05 | -0,315 | 0,235 | 1,62E-01 | -0,507 | 0,118 | 1,02E-03 |
| FBG1n/G1n | ana | 0,581 | 0,137 | 2,22E-05 | 0,268 | 0,234 | 2,33E-01 | 0,501 | 0,118 | 1,33E-03 |
| GP23 | ana | -0,492 | 0,142 | 4,92E-04 | -0,509 | 0,228 | 2,12E-02 | -0,497 | 0,121 | 1,86E-03 |
| GP6n | ana | 0,494 | 0,129 | 1,24E-04 | 0,346 | 0,220 | 1,01E-01 | 0,456 | 0,111 | 1,95E-03 |
| GP6 | ana | 0,462 | 0,130 | 3,47E-04 | 0,352 | 0,220 | 9,59E-02 | 0,433 | 0,112 | 4,40E-03 |
| Fn | ana | -0,479 | 0,136 | 4,00E-04 | -0,301 | 0,232 | 1,75E-01 | -0,433 | 0,117 | 8,18E-03 |
| FS1/FS2 | ana | 0,392 | 0,143 | 5,42E-03 | 0,545 | 0,219 | 1,05E-02 | 0,438 | 0,120 | 8,18E-03 |
| GP10 | ana | 0,435 | 0,139 | 1,64E-03 | 0,284 | 0,234 | 2,05E-01 | 0,395 | 0,120 | 2,30E-02 |
| FG2n/G2n | ana | -0,488 | 0,135 | 2,76E-04 | -0,056 | 0,232 | 8,00E-01 | -0,379 | 0,117 | 2,38E-02 |
| GP20 | ana | -0,431 | 0,147 | 3,06E-03 | -0,332 | 0,231 | 1,34E-01 | -0,403 | 0,124 | 2,38E-02 |
| BG2n/(FG2n + FBG2n) | ana | 0,448 | 0,134 | 7,43E-04 | 0,133 | 0,233 | 5,50E-01 | 0,370 | 0,116 | 2,49E-02 |
| GP14n | ana | -0,448 | 0,131 | 5,82E-04 | -0,082 | 0,218 | 6,94E-01 | -0,351 | 0,112 | 2,82E-02 |
| GP14 | ana | -0,465 | 0,131 | 3,50E-04 | -0,015 | 0,220 | 9,42E-01 | -0,347 | 0,112 | 3,01E-02 |
| G0n | ana | 0,400 | 0,135 | 2,80E-03 | 0,181 | 0,224 | 3,99E-01 | 0,342 | 0,116 | 4,13E-02 |
| FBG2S1/(FBG2+FBG2S1+FBG2S2) | la | 0,612 | 0,179 | 5,59E-04 | 0,441 | 0,343 | 1,81E-01 | 0,576 | 0,158 | 8,72E-03 |
| FBS1/(FS1+FBS1) | la | 0,553 | 0,173 | 1,27E-03 | 0,533 | 0,330 | 9,28E-02 | 0,548 | 0,153 | 9,96E-03 |
| FBS1/FS1 | la | 0,553 | 0,173 | 1,27E-03 | 0,533 | 0,330 | 9,28E-02 | 0,548 | 0,153 | 9,96E-03 |
| FBStotal/FStotal | la | 0,481 | 0,171 | 4,53E-03 | 0,497 | 0,329 | 1,16E-01 | 0,484 | 0,152 | 2,49E-02 |
| GP12n | la | -0,431 | 0,182 | 1,68E-02 | -0,765 | 0,332 | 1,73E-02 | -0,508 | 0,160 | 2,49E-02 |
| GP12 | la | -0,429 | 0,183 | 1,74E-02 | -0,772 | 0,338 | 1,83E-02 | -0,507 | 0,161 | 2,63E-02 |
| GP1 | la | 0,491 | 0,181 | 6,18E-03 | 0,389 | 0,337 | 2,30E-01 | 0,468 | 0,160 | 4,29E-02 |
| GP1n | la | 0,486 | 0,182 | 6,93E-03 | 0,376 | 0,340 | 2,49E-01 | 0,462 | 0,160 | 4,82E-02 |
| FBG2S1/(FBG2+FBG2S1+FBG2S2) | la_val | 0,117 | 0,027 | 1,85E-05 | 0,524 | 0,300 | 6,94E-02 | 0,120 | 0,027 | 1,00E-03 |
| FBS1/(FS1+FBS1) | la_val | 0,107 | 0,026 | 4,74E-05 | 0,475 | 0,290 | 8,88E-02 | 0,110 | 0,026 | 1,42E-03 |
| FBS1/FS1 | la_val | 0,107 | 0,026 | 4,74E-05 | 0,475 | 0,290 | 8,88E-02 | 0,110 | 0,026 | 1,42E-03 |
| FBStotal/FStotal | la_val | 0,094 | 0,026 | 3,32E-04 | 0,400 | 0,290 | 1,50E-01 | 0,096 | 0,026 | 8,18E-03 |
| GP14 | la_val | -0,083 | 0,025 | 1,02E-03 | -0,285 | 0,282 | 2,90E-01 | -0,085 | 0,025 | 2,11E-02 |
| FBG2n/G2n | la_val | 0,087 | 0,027 | 1,11E-03 | 0,112 | 0,288 | 6,85E-01 | 0,088 | 0,027 | 2,38E-02 |
| FBG2n /(FG2n + FBG2n ) | la_val | 0,086 | 0,027 | 1,12E-03 | 0,010 | 0,290 | 9,71E-01 | 0,085 | 0,027 | 2,43E-02 |
| FBG2n/FG2n | la_val | 0,086 | 0,027 | 1,12E-03 | 0,010 | 0,290 | 9,71E-01 | 0,085 | 0,027 | 2,43E-02 |
| GP14n | la_val | -0,080 | 0,026 | 1,67E-03 | -0,328 | 0,279 | 2,20E-01 | -0,082 | 0,025 | 2,43E-02 |
| G0n | la_val | 0,080 | 0,026 | 1,90E-03 | 0,451 | 0,286 | 1,01E-01 | 0,084 | 0,026 | 2,43E-02 |
| GP4n | la_val | 0,079 | 0,027 | 2,79E-03 | 0,479 | 0,290 | 8,57E-02 | 0,082 | 0,026 | 2,96E-02 |
| GP19 | la_val | 0,086 | 0,028 | 2,37E-03 | 0,276 | 0,303 | 3,41E-01 | 0,087 | 0,028 | 3,04E-02 |
| G2n | la_val | -0,076 | 0,026 | 3,01E-03 | -0,399 | 0,279 | 1,37E-01 | -0,079 | 0,026 | 3,15E-02 |
| FG2n/(BG2n + FBG2n) | la_val | -0,081 | 0,026 | 2,06E-03 | 0,010 | 0,291 | 9,71E-01 | -0,080 | 0,026 | 3,30E-02 |
| GP4 | la_val | 0,075 | 0,027 | 4,75E-03 | 0,487 | 0,289 | 7,93E-02 | 0,078 | 0,027 | 4,26E-02 |
| GP18 | la_val | -0,073 | 0,026 | 4,09E-03 | -0,288 | 0,289 | 2,98E-01 | -0,074 | 0,025 | 4,39E-02 |
| GP6n | la_val | 0,072 | 0,025 | 4,26E-03 | 0,195 | 0,287 | 4,78E-01 | 0,073 | 0,025 | 4,82E-02 |
| FS1/FS2 | rnp | 0,505 | 0,122 | 3,56E-05 | 0,192 | 0,207 | 3,32E-01 | 0,424 | 0,105 | 2,46E-03 |
| FG2S1/(FG2+FG2S1+FG2S2) | rnp | 0,322 | 0,127 | 1,06E-02 | 0,450 | 0,208 | 2,55E-02 | 0,357 | 0,109 | 2,38E-02 |
| FBG2S1/(FBG2+FBG2S1+FBG2S2) | rnp | -0,249 | 0,126 | 4,52E-02 | -0,631 | 0,207 | 1,84E-03 | -0,353 | 0,108 | 2,38E-02 |
| GP9n | rnp | -0,127 | 0,126 | 3,05E-01 | -0,869 | 0,195 | 8,93E-06 | -0,345 | 0,106 | 2,38E-02 |
| GP9 | rnp | -0,117 | 0,128 | 3,50E-01 | -0,853 | 0,194 | 1,19E-05 | -0,339 | 0,107 | 2,49E-02 |
| FG2S2/(FG2+FG2S1+FG2S2) | rnp | -0,367 | 0,121 | 2,25E-03 | -0,187 | 0,205 | 3,42E-01 | -0,320 | 0,104 | 3,12E-02 |
| GP21 | rnp | -0,307 | 0,129 | 1,57E-02 | -0,382 | 0,210 | 5,94E-02 | -0,328 | 0,110 | 3,80E-02 |
| FS1/FS2 | rnp_val | 0,074 | 0,021 | 3,63E-04 | 0,194 | 0,148 | 1,72E-01 | 0,076 | 0,021 | 8,18E-03 |
| GP9n | rnp_val | -0,062 | 0,021 | 2,65E-03 | -0,587 | 0,141 | 3,12E-05 | -0,074 | 0,021 | 1,07E-02 |
| FG2S1/(FG2+FG2S1+FG2S2) | rnp_val | 0,069 | 0,021 | 1,14E-03 | 0,280 | 0,150 | 5,29E-02 | 0,073 | 0,021 | 1,48E-02 |
| GP1 | rnp_val | 0,073 | 0,021 | 4,89E-04 | -0,055 | 0,152 | 7,07E-01 | 0,071 | 0,021 | 1,91E-02 |
| GP23 | rnp_val | -0,069 | 0,021 | 9,03E-04 | -0,088 | 0,154 | 5,50E-01 | -0,070 | 0,021 | 2,11E-02 |
| GP1n | rnp_val | 0,073 | 0,021 | 5,81E-04 | -0,097 | 0,153 | 5,10E-01 | 0,069 | 0,021 | 2,30E-02 |
| GP9 | rnp_val | -0,057 | 0,021 | 7,34E-03 | -0,535 | 0,143 | 1,49E-04 | -0,067 | 0,021 | 2,49E-02 |
| FG2S1/(FG2+FG2S1+FG2S2) | ro | 0,358 | 0,125 | 3,86E-03 | 0,282 | 0,216 | 1,74E-01 | 0,339 | 0,108 | 2,79E-02 |
| GP20 | ro | -0,317 | 0,127 | 1,15E-02 | -0,309 | 0,217 | 1,38E-01 | -0,315 | 0,110 | 4,82E-02 |
| GP1 | sm | 0,362 | 0,131 | 5,26E-03 | 0,385 | 0,290 | 1,67E-01 | 0,366 | 0,119 | 3,15E-02 |
| GP9n | sm | -0,369 | 0,129 | 3,84E-03 | -0,290 | 0,294 | 3,04E-01 | -0,356 | 0,118 | 3,50E-02 |
| GP1n | sm | 0,354 | 0,131 | 6,53E-03 | 0,317 | 0,293 | 2,59E-01 | 0,348 | 0,120 | 4,64E-02 |
| FBS2/(FS2+FBS2) | sm_val | 0,074 | 0,020 | 2,28E-04 | 0,038 | 0,185 | 8,30E-01 | 0,073 | 0,020 | 8,18E-03 |
| FBS2/FS2 | sm_val | 0,074 | 0,020 | 2,28E-04 | 0,038 | 0,185 | 8,30E-01 | 0,073 | 0,020 | 8,18E-03 |
| FBG2n/G2n | sm_val | 0,065 | 0,020 | 1,27E-03 | 0,071 | 0,179 | 6,78E-01 | 0,065 | 0,020 | 2,43E-02 |
| FG2S1/(FG2+FG2S1+FG2S2) | sm_val | 0,062 | 0,021 | 3,02E-03 | 0,145 | 0,185 | 4,14E-01 | 0,063 | 0,021 | 3,55E-02 |
| GP5 | arthritis | 0,339 | 0,151 | 2,27E-02 | 0,408 | 0,250 | 9,13E-02 | 0,358 | 0,129 | 5,55E-02 |
| FBGS/(FB+FBG+FBGS) | convulsions | -0,737 | 0,196 | 1,56E-04 | -0,422 | 0,473 | 3,50E-01 | -0,691 | 0,181 | 3,74E-03 |
| GP23 | convulsions | -0,690 | 0,196 | 3,94E-04 | -0,470 | 0,476 | 3,02E-01 | -0,658 | 0,181 | 6,67E-03 |
| FBGS/(FBG+FBGS) | convulsions | -0,767 | 0,198 | 1,03E-04 | -0,004 | 0,477 | 9,93E-01 | -0,655 | 0,183 | 7,51E-03 |
| GP6 | convulsions | 0,406 | 0,183 | 2,44E-02 | 1,172 | 0,441 | 6,27E-03 | 0,518 | 0,169 | 3,38E-02 |
| FBn | convulsions | 0,453 | 0,190 | 1,60E-02 | 1,026 | 0,461 | 2,14E-02 | 0,536 | 0,176 | 3,45E-02 |
| FBG1n/G1n | convulsions | 0,486 | 0,193 | 1,09E-02 | 0,834 | 0,474 | 6,76E-02 | 0,536 | 0,179 | 3,78E-02 |
| FG1n/G1n | convulsions | -0,478 | 0,192 | 1,15E-02 | -0,831 | 0,474 | 6,89E-02 | -0,528 | 0,178 | 3,82E-02 |
| GP6n | convulsions | 0,384 | 0,183 | 3,32E-02 | 1,181 | 0,441 | 5,87E-03 | 0,502 | 0,169 | 3,82E-02 |
| FBn/Fn | convulsions | 0,448 | 0,190 | 1,70E-02 | 0,948 | 0,463 | 3,38E-02 | 0,520 | 0,176 | 3,82E-02 |
| FBn/Fn total | convulsions | 0,448 | 0,190 | 1,70E-02 | 0,948 | 0,463 | 3,38E-02 | 0,520 | 0,176 | 3,82E-02 |
| Fn/(Bn + FBn) | convulsions | -0,441 | 0,190 | 1,88E-02 | -0,941 | 0,463 | 3,54E-02 | -0,513 | 0,176 | 4,05E-02 |
| FS1/FS2 | convulsions | 0,622 | 0,196 | 1,37E-03 | -0,103 | 0,463 | 8,16E-01 | 0,512 | 0,180 | 4,73E-02 |
| GP10 | convulsions | 0,481 | 0,194 | 1,19E-02 | 0,548 | 0,478 | 2,32E-01 | 0,491 | 0,179 | 5,59E-02 |
| GP10 | duration | 0,290 | 0,068 | 2,20E-05 | 0,298 | 0,106 | 3,97E-03 | 0,292 | 0,057 | 1,73E-04 |
| FBn | duration | 0,306 | 0,067 | 4,94E-06 | 0,173 | 0,107 | 9,34E-02 | 0,269 | 0,057 | 4,94E-04 |
| GP10n | duration | 0,247 | 0,069 | 3,59E-04 | 0,311 | 0,106 | 2,58E-03 | 0,266 | 0,058 | 4,94E-04 |
| FBn/Fn | duration | 0,310 | 0,067 | 3,71E-06 | 0,123 | 0,108 | 2,37E-01 | 0,258 | 0,057 | 4,94E-04 |
| FBn/Fn total | duration | 0,310 | 0,067 | 3,71E-06 | 0,123 | 0,108 | 2,37E-01 | 0,258 | 0,057 | 4,94E-04 |
| Fn/(Bn + FBn) | duration | -0,310 | 0,067 | 3,70E-06 | -0,117 | 0,108 | 2,61E-01 | -0,257 | 0,057 | 4,94E-04 |
| GP1n | duration | -0,124 | 0,073 | 8,33E-02 | -0,502 | 0,097 | 3,38E-07 | -0,261 | 0,058 | 4,94E-04 |
| FBG1n/G1n | duration | 0,282 | 0,068 | 3,72E-05 | 0,190 | 0,108 | 6,90E-02 | 0,256 | 0,058 | 5,96E-04 |
| FG1n/G1n | duration | -0,308 | 0,067 | 5,14E-06 | -0,054 | 0,110 | 6,13E-01 | -0,239 | 0,058 | 1,57E-03 |
| GP1 | duration | -0,107 | 0,073 | 1,35E-01 | -0,465 | 0,098 | 2,57E-06 | -0,234 | 0,058 | 2,47E-03 |
| Fn | duration | -0,317 | 0,067 | 2,26E-06 | 0,032 | 0,109 | 7,61E-01 | -0,222 | 0,057 | 3,11E-03 |
| FBS2/(FS2+FBS2) | duration | 0,276 | 0,069 | 6,12E-05 | 0,088 | 0,108 | 3,96E-01 | 0,221 | 0,058 | 3,74E-03 |
| FBS2/FS2 | duration | 0,276 | 0,069 | 6,12E-05 | 0,088 | 0,108 | 3,96E-01 | 0,221 | 0,058 | 3,74E-03 |
| FBG0n/G0n | duration | 0,237 | 0,070 | 5,94E-04 | 0,133 | 0,108 | 2,00E-01 | 0,206 | 0,058 | 8,52E-03 |
| FG0n/G0n | duration | -0,269 | 0,069 | 1,04E-04 | 0,010 | 0,109 | 9,27E-01 | -0,188 | 0,059 | 2,23E-02 |
| GP6n | duration | 0,210 | 0,065 | 1,24E-03 | 0,029 | 0,105 | 7,75E-01 | 0,159 | 0,056 | 4,48E-02 |
| GP6 | duration | 0,202 | 0,065 | 1,81E-03 | 0,027 | 0,105 | 7,88E-01 | 0,153 | 0,056 | 5,56E-02 |
| GP20 | duration | -0,072 | 0,074 | 3,26E-01 | -0,349 | 0,104 | 6,24E-04 | -0,166 | 0,060 | 5,59E-02 |
| GP14 | pericarditis | -0,609 | 0,150 | 4,69E-05 | -0,214 | 0,238 | 3,48E-01 | -0,497 | 0,127 | 3,11E-03 |
| GP6n | pericarditis | 0,688 | 0,148 | 3,69E-06 | -0,029 | 0,242 | 8,99E-01 | 0,493 | 0,126 | 3,11E-03 |
| GP14n | pericarditis | -0,610 | 0,150 | 4,57E-05 | -0,111 | 0,237 | 6,23E-01 | -0,467 | 0,127 | 5,65E-03 |
| GP6 | pericarditis | 0,668 | 0,149 | 7,16E-06 | -0,097 | 0,242 | 6,76E-01 | 0,459 | 0,127 | 6,67E-03 |
| G2n | pericarditis | -0,578 | 0,152 | 1,32E-04 | -0,117 | 0,237 | 6,05E-01 | -0,444 | 0,128 | 1,02E-02 |
| G0n | pericarditis | 0,617 | 0,154 | 5,75E-05 | -0,017 | 0,246 | 9,41E-01 | 0,439 | 0,130 | 1,46E-02 |
| FG2n/G2n | pericarditis | -0,563 | 0,156 | 2,81E-04 | -0,072 | 0,253 | 7,66E-01 | -0,428 | 0,133 | 2,22E-02 |
| BG2n/(FG2n + FBG2n) | pericarditis | 0,529 | 0,154 | 5,70E-04 | 0,083 | 0,252 | 7,29E-01 | 0,407 | 0,132 | 3,31E-02 |
| GP18 | pericarditis | -0,509 | 0,150 | 6,44E-04 | -0,053 | 0,244 | 8,19E-01 | -0,384 | 0,128 | 3,78E-02 |
| GP4n | pericarditis | 0,559 | 0,157 | 3,36E-04 | -0,043 | 0,248 | 8,57E-01 | 0,387 | 0,133 | 4,05E-02 |
| GP9n | pericarditis | -0,615 | 0,162 | 1,34E-04 | 0,157 | 0,252 | 5,15E-01 | -0,390 | 0,136 | 4,48E-02 |
| FG2n total /G2n | pericarditis | -0,505 | 0,161 | 1,62E-03 | -0,050 | 0,256 | 8,38E-01 | -0,375 | 0,137 | 5,59E-02 |
| GP2n | pericarditis | 0,446 | 0,158 | 4,27E-03 | 0,160 | 0,242 | 4,89E-01 | 0,361 | 0,132 | 5,59E-02 |
| GP4 | pericarditis | 0,548 | 0,157 | 4,45E-04 | -0,101 | 0,247 | 6,70E-01 | 0,361 | 0,133 | 5,60E-02 |
| GP2 | proteinuria | 0,421 | 0,120 | 4,00E-04 | 0,451 | 0,203 | 2,16E-02 | 0,429 | 0,103 | 1,57E-03 |
| GP2n | proteinuria | 0,418 | 0,120 | 4,60E-04 | 0,449 | 0,204 | 2,29E-02 | 0,426 | 0,103 | 1,66E-03 |
| FG0n total/G0n | proteinuria | -0,346 | 0,127 | 5,75E-03 | -0,388 | 0,210 | 5,51E-02 | -0,357 | 0,108 | 1,81E-02 |
| GP6n | proteinuria | 0,330 | 0,117 | 4,34E-03 | 0,255 | 0,207 | 2,00E-01 | 0,312 | 0,102 | 3,38E-02 |
| GP6 | proteinuria | 0,318 | 0,117 | 5,96E-03 | 0,273 | 0,207 | 1,69E-01 | 0,307 | 0,102 | 3,69E-02 |
| GP9n | proteinuria | -0,472 | 0,124 | 1,34E-04 | 0,166 | 0,217 | 4,26E-01 | -0,315 | 0,108 | 4,05E-02 |
| FG1n total/G1n | proteinuria | -0,292 | 0,125 | 1,83E-02 | -0,372 | 0,216 | 7,48E-02 | -0,312 | 0,108 | 4,48E-02 |
| FG2n/G2n | proteinuria | -0,281 | 0,121 | 1,90E-02 | -0,354 | 0,216 | 8,79E-02 | -0,299 | 0,106 | 4,84E-02 |
| GP18 | proteinuria | -0,265 | 0,117 | 2,16E-02 | -0,341 | 0,206 | 8,61E-02 | -0,283 | 0,102 | 5,28E-02 |


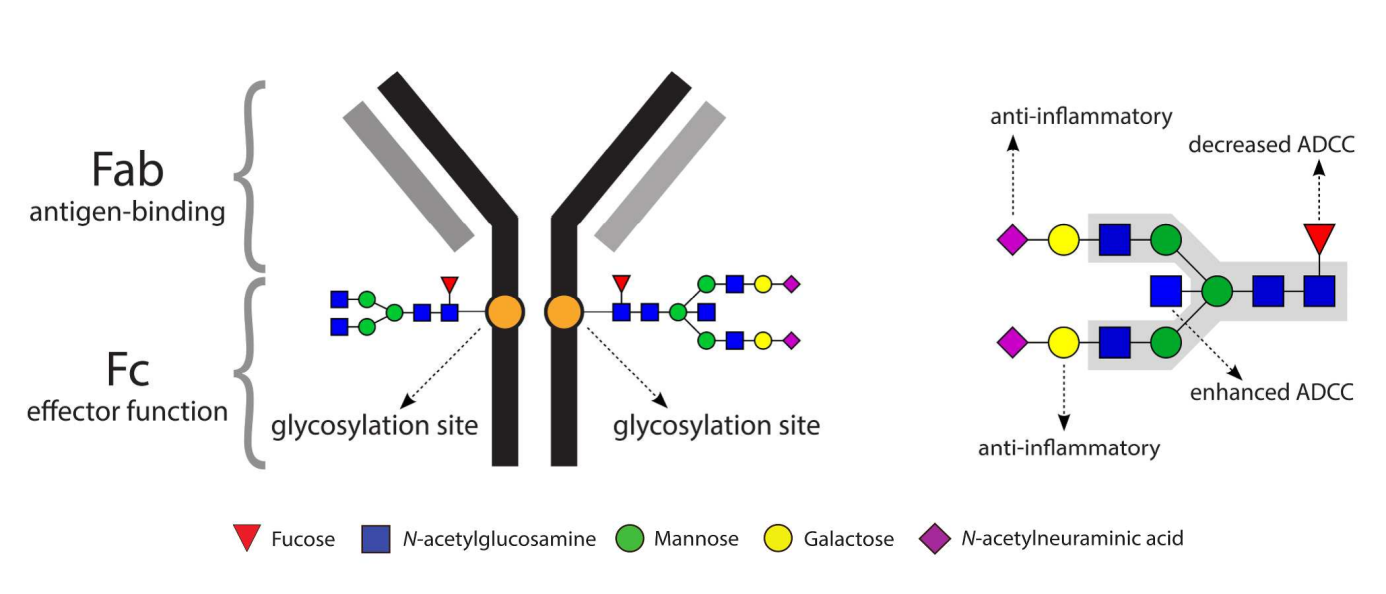


**Supplementary Figure 1. Functional relevance of IgG glycosylation**. Glycans play an important functional role on all immunoglobulins. When attached to the conserved Asn297 in IgG heavy chain they significantly participate in the structure and the overall conformation of IgG conserved region. Deglycosylated IgG can not activate effector functions, while the attachment of different glycans to this site promotes binding to different Fc receptors and in this way modulate effector functions (45). Between 15% and 20% of IgG also bears glycans on the variable region of IgG, but functional relevance of these glycans is not known.


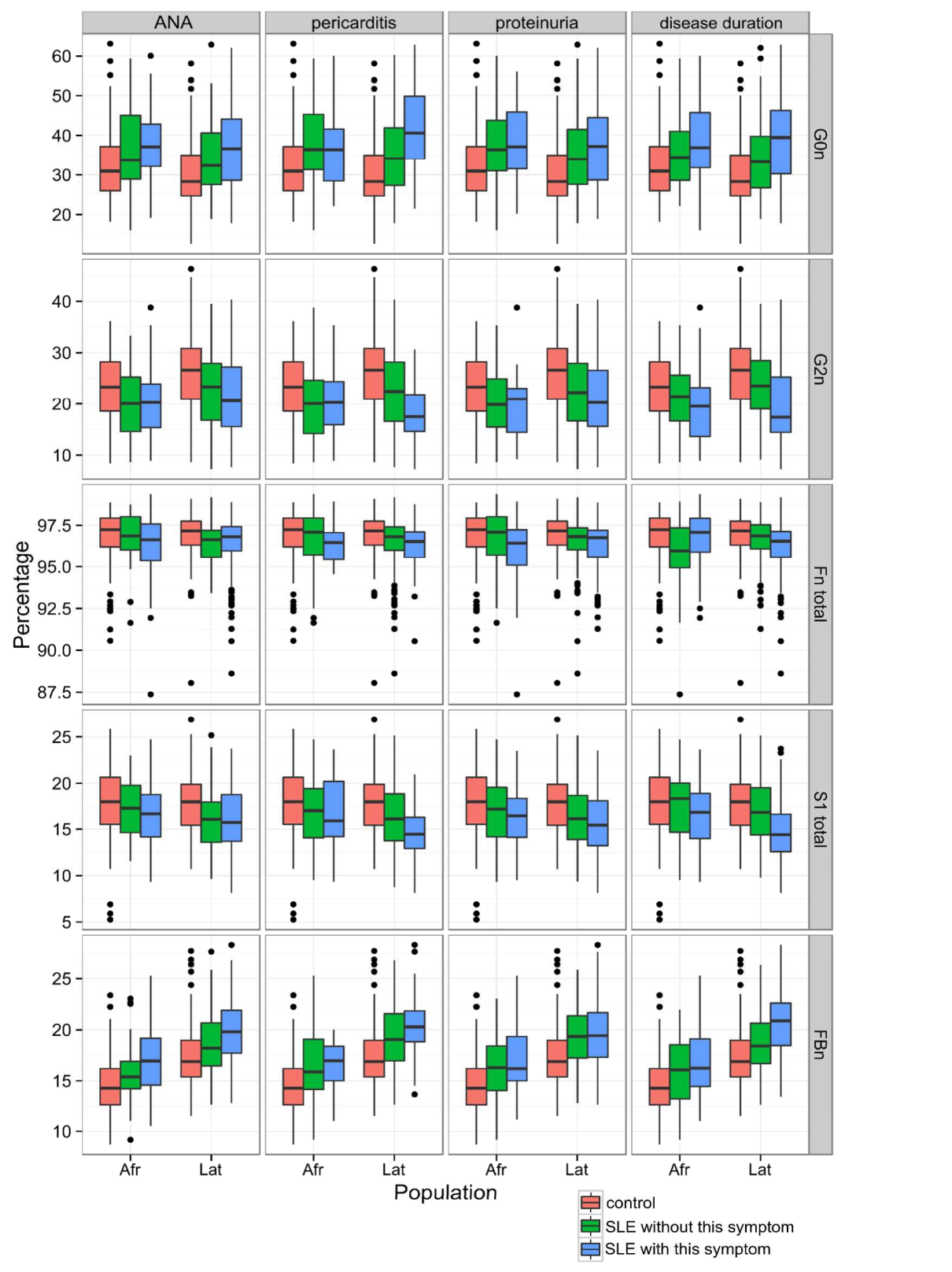


**Supplementary Figure 2. Association of glycosylation changes with SLE related clinical features in Afro- Caribbean (n = 110) and Latino American (n = 261) cohorts.** Changes in glycosylation were more pronounced in patients with positive ANA test, pericarditis, proteinuria or in patients with disease duration of over 8 years. While bisecting GlcNAc was increased in patients with severe symptoms, sialylation, galactosylation and fucosylation were in most cases decreased.

* G0n = Proportion of agalactosylated structures in neutral glycans; G2n = Proportion of digalactosylated structures in neutral glycans; Fn total = Proportion of fucosylated structures in neutral glycans; S1 total = Proportion of monosialylated structures in total IgG glycans; FBn = Proportion of fucosylated (with bisecting GlcNAc) structures in total neutral IgG glycans.


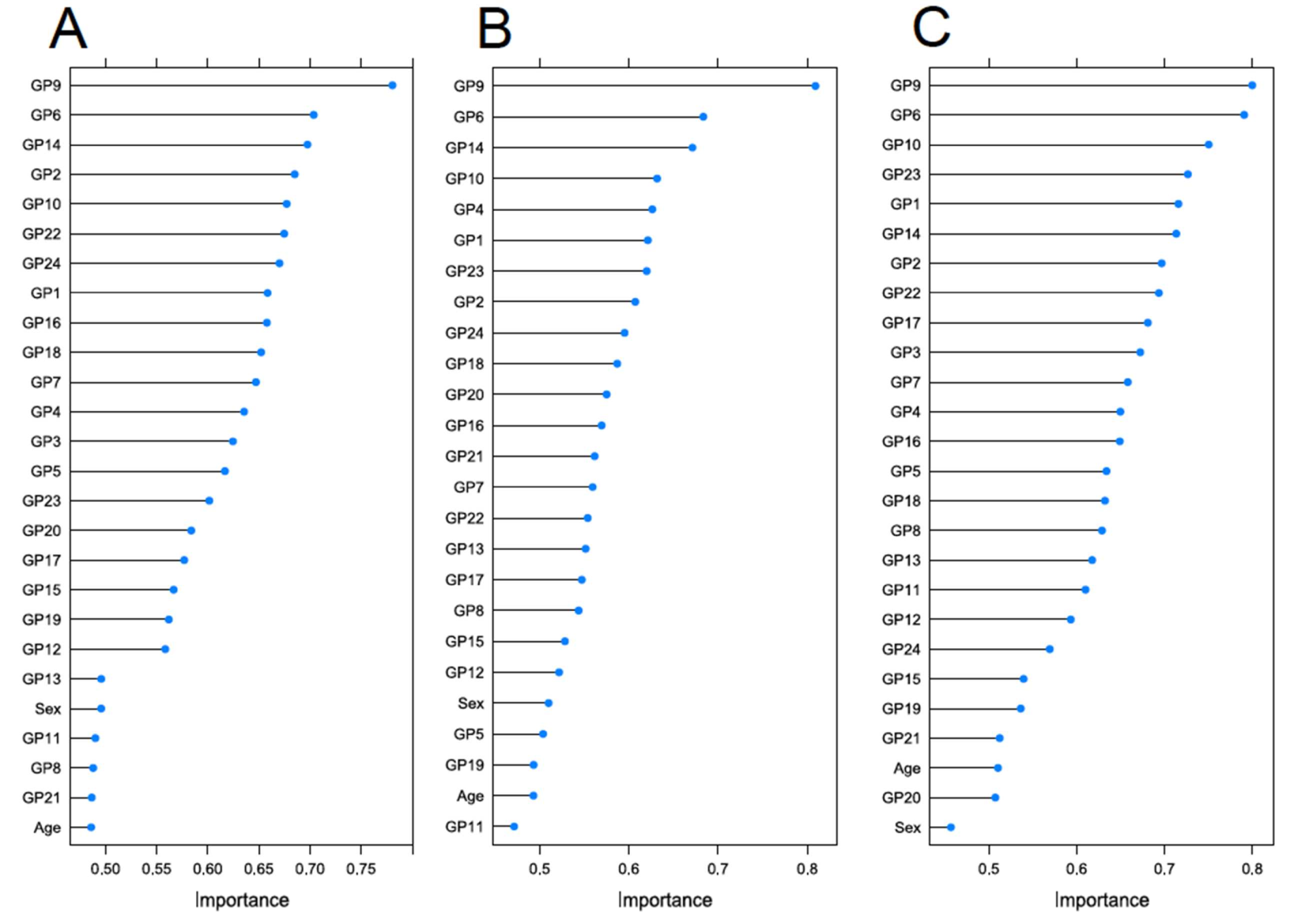


**Supplementary Figure 3. Variable importance in glycan based SLE discriminative models.** Predictive power of each individual glycan trait was evaluated by ROC curve analysis on (A) Afro-Caribbean, (B) Latin American and (C) Chinese cohort.
